# Non-*Leishmania* Parasite in Fatal Visceral Leishmaniasis–Like Disease, Brazil

**DOI:** 10.3201/eid2511.181548

**Published:** 2019-11

**Authors:** Sandra R. Maruyama, Alynne K.M. de Santana, Nayore T. Takamiya, Talita Y. Takahashi, Luana A. Rogerio, Caio A.B. Oliveira, Cristiane M. Milanezi, Viviane A. Trombela, Angela K. Cruz, Amélia R. Jesus, Aline S. Barreto, Angela M. da Silva, Roque P. Almeida, José M. Ribeiro, João S. Silva

**Affiliations:** Universidade Federal de São Carlos, São Carlos, Brazil (S.R. Maruyama, N.T. Takamiya, T.Y. Takahashi, L.A. Rogerio, C.A.B. Oliveira);; Universidade Federal de Sergipe, Aracaju, Brazil (A.K.M. de Santana, A.R. Jesus, A.S. Barreto, A.M. da Silva, R.P. Almeida);; Universidade de São Paulo, Ribeirão Preto, Brazil (C.M. Milanezi, V.A. Trombela, A.K. Cruz);; National Institutes of Health, Rockville, Maryland, USA (J.M. Ribeiro);; Fundação Oswaldo Cruz Bi-institucional, Ribeirão Preto (J.S. Silva)

**Keywords:** Visceral leishmaniasis–like, Crithidia-related, genome sequencing, parasites, Brazil, whole-genome sequencing, sand flies, parasites, vector-borne infections

## Abstract

Through whole-genome sequencing analysis, we identified non-*Leishmania* parasites isolated from a man with a fatal visceral leishmaniasis–like illness in Brazil. The parasites infected mice and reproduced the patient’s clinical manifestations. Molecular epidemiologic studies are needed to ascertain whether a new infectious disease is emerging that can be confused with leishmaniasis.

Leishmaniases are caused by ≈20 *Leishmania* species transmitted to humans through sand-fly bites and have been classified into 3 main forms: cutaneous leishmaniasis, mucocutaneous or mucosal leishmaniasis, and visceral leishmaniasis (VL; also known as kala-azar) (*1**,**2*). VL is the most severe form of the disease and can be fatal if misdiagnosed or untreated (*3*). Cases of VL in Brazil account for >90% of annual reported cases in Latin America (*4*), where the causative species is *L. infantum*.

Since 1980, sporadic co-infections of *Leishmania* with apparently monoxenous trypanosomatids have been described (Kaufer et al. [*5*]); these reports are sometimes associated with immunocompromised hosts. More recently, coinfections with *Crithidia*-like (*6*) or *Leptomonas-*like (*7*) parasites have been reported. Whether these co-infections are occasional findings or are evidence for new parasites with the potential to threaten public health remains uncertain. To investigate this problem, we performed whole-genome sequencing of 2 clinical isolates from a patient with a fatal illness with clinical characteristics similar to those of VL.

## The Study

During 2011–2012, we characterized 2 parasite strains, LVH60 and LVH60a, isolated from an HIV-negative man when he was 64 years old and 65 years old (Table; Appendix). Treatment-refractory VL-like disease developed in the man; signs and symptoms consisted of weight loss, fever, anemia, low leukocyte and platelet counts, and severe liver and spleen enlargements. VL was confirmed by light microscopic examination of amastigotes in bone marrow aspirates and promastigotes in culture upon parasite isolation and by positive rK39 serologic test results. Three courses of liposomal amphotericin B resulted in no response. At the third hospital admission, the illness resembled diffuse cutaneous leishmaniasis, in which several disseminated papular skin lesions were observed (Appendix Figure 1, panel A), and a skin biopsy revealed macrophages filled with amastigotes (Appendix Figure 1, panel B), which his liver biopsy results also showed (Appendix Figure 1, panel C). During this third admission, the LVH60a strain was isolated from the skin. Dermal lesions known as post–kala-azar dermal leishmaniasis (PKDL) have rarely been reported in Brazil (*13*), and the clinical aspect of the disseminated papular skin lesions on this patient differed from the clinical presentation of PKDL. Because his illness did not respond to therapy, the patient underwent splenectomy. He died of disease and surgical complications.

**Table:** Non-*Leishmani*a parasites isolated from 2 patients with visceral leishmaniasis–like illness used for whole-genome sequencing, Brazil*

Clinical isolate	Year isolated	Tissue source	Patient age, y/sex	Treatment	Recidivism	Healing time	Serologic test (rK39)	MLEE	Experimental assays
LVH60	2011	BM	64/M	Liposomal amphotericin B	Yes, 3	Fatal case	Positive	Inconclusive	Mouse infection (this study)
LVH60a (DPSLs)	2012	SL	65/M	Liposomal amphotericin B	Yes, 3	Fatal case	Positive	Inconclusive	Mouse infection (this study)
HU-UFS14	2009	BM	15/M	Antimony, amphotericin B	NA	NA	Positive	*L. infantum*	NO- and antimony-resistant (*8*); murine model of infection (*9*–*12*).

*BM, bone marrow; DPSL, disseminated popular skin lesions; MLEE, multilocus enzyme electrophoresis; NA, not available or not applicable; NO, nitrite oxide; SL, skin lesion.

**Figure 1:**
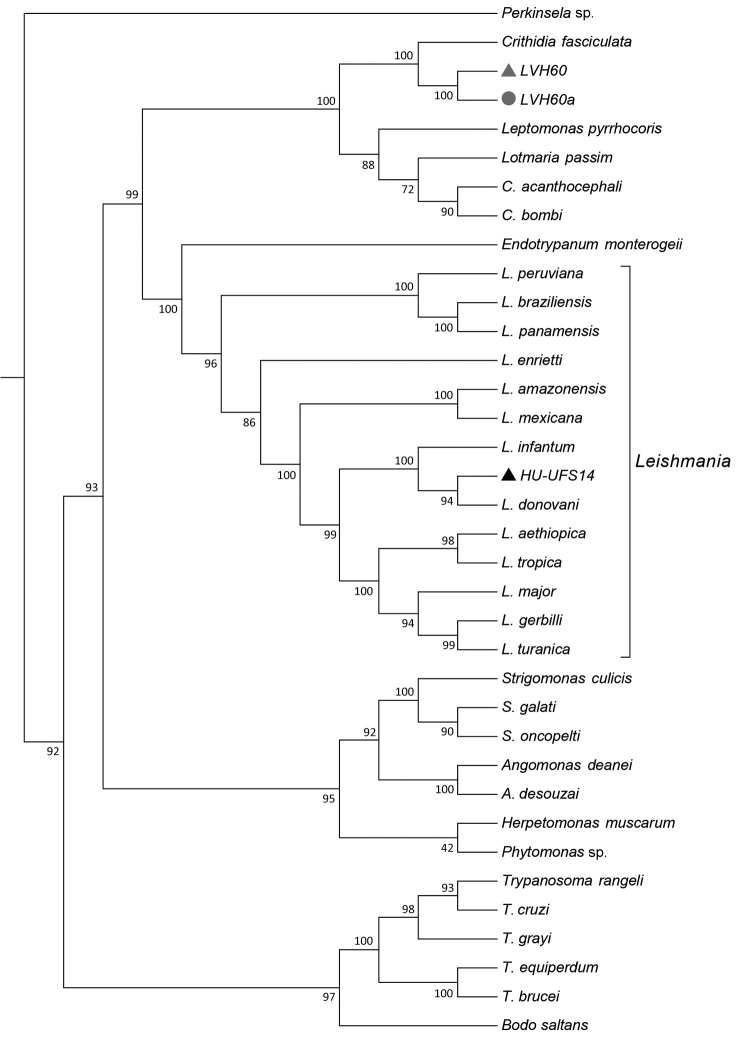
Phylogenomic analysis of genomewide orthologous coding sequences from LVH60 and LVH60a clinical isolates from a 64-year-old man with fatal visceral leishmaniasis–like illness, Brazil, and 33 Trypanosomatida species. Dendrogram shows the genetic relationships among all species investigated in the current study. Hierarchical clustering was performed with a set of ≈6,400 orthologous genes across 33 trypanosomatids, designated as the total orthologous median matrix. HU-UFS14 (black triangle; *L. infantum* laboratory reference strain) is placed in the same branch with *L. infantum* and *L. donovani*, whereas the LVH60 and LVH60a clinical isolates are placed in sister positions with *Crithidia fasciculata*. LVH60 was isolated from bone marrow (gray triangle), LVH60a from a skin lesion (gray circle) biopsy, both from the same patient. Numbers next to the branches represent the percentages of approximate unbiased support probabilities for 10,000 bootstraps, calculated using the pvclust R package (https://cran.r-project.org/web/packages/pvclust). Branch relationships were defined by their median amino acid evolutionary distance (Appendix).

We used cryopreserved parasite stocks isolated from bone marrow (LVH60) and skin lesions (LVH60a) to obtain promastigotes for DNA isolation. We obtained clonal colonies and analyzed them to confirm the homogeneity of parasite cultures. For species identification, we amplified the small subunit rRNA (SSU rRNA), ribosomal internal transcribed spacer 1 (ITS1) regions, and glyceraldehyde 3-phosphate dehydrogenase gene (GAPDH) by PCR, sequenced them, and analyzed them. We used a laboratory reference *L. infantum* strain (HU-UFS14) used in experimental infections elsewhere (*9*–*12*) as control. A PCR using primers for HSP70 gene (specific to discriminate *Leishmania* species [*14*]) resulted in no amplification. Amplicon sequence analyses of SSU rRNA, ITS1, and GAPDH revealed that the LVH60 and LVH60a strains are more closely related to *Crithidia fasciculata* than to *Leishmania*. Only the HU-UFS14 clustered within the *Leishmania* group on a branch composed of *L. infantum* and *L. donovani*.

To characterize the organisms LVH60 and LVH60a, we determined their complete genome sequences with >400× coverage. We assembled the reads into ≈4,500 scaffolds. More than 9,000 coding sequences were deduced per isolate. Only HU-UFS14 presented a predicted haploid genome size similar to that of a known *Leishmania* species (≈33 Mb).

To ascertain the phylogenetic relationships between these isolates, we developed a comprehensive strategy to compare all available trypanosomatid orthologous proteins, in which we calculated a pairwise distance matrix based on the median distance of orthologous genes found using the RSD algorithm (S.R. Maruyama et al., unpub. data). We identified an average of 6,093 orthologs for all considered pairs. Corroborating the phylogenies of single sequences (SSU rRNA, ITS1, and GAPDH), both clinical isolates (except HU-UFS14) clustered apart from the *Leishmania* clade (Figure 1), fitting into another Leishmaniinae subfamily group composed of the monoxenous genera *Leptomonas*, *Lotmaria*, and *Crithidia*, which infect only insect hosts (*5*). These results revealed that the LVH60 and LVH60a isolates do not belong to the *Leishmania* genus. Instead, these isolates form a robust clade including *C. fasciculata* but excluding 2 other *Crithidia* and *Lotmaria* bee parasites.

**Figure 2:**
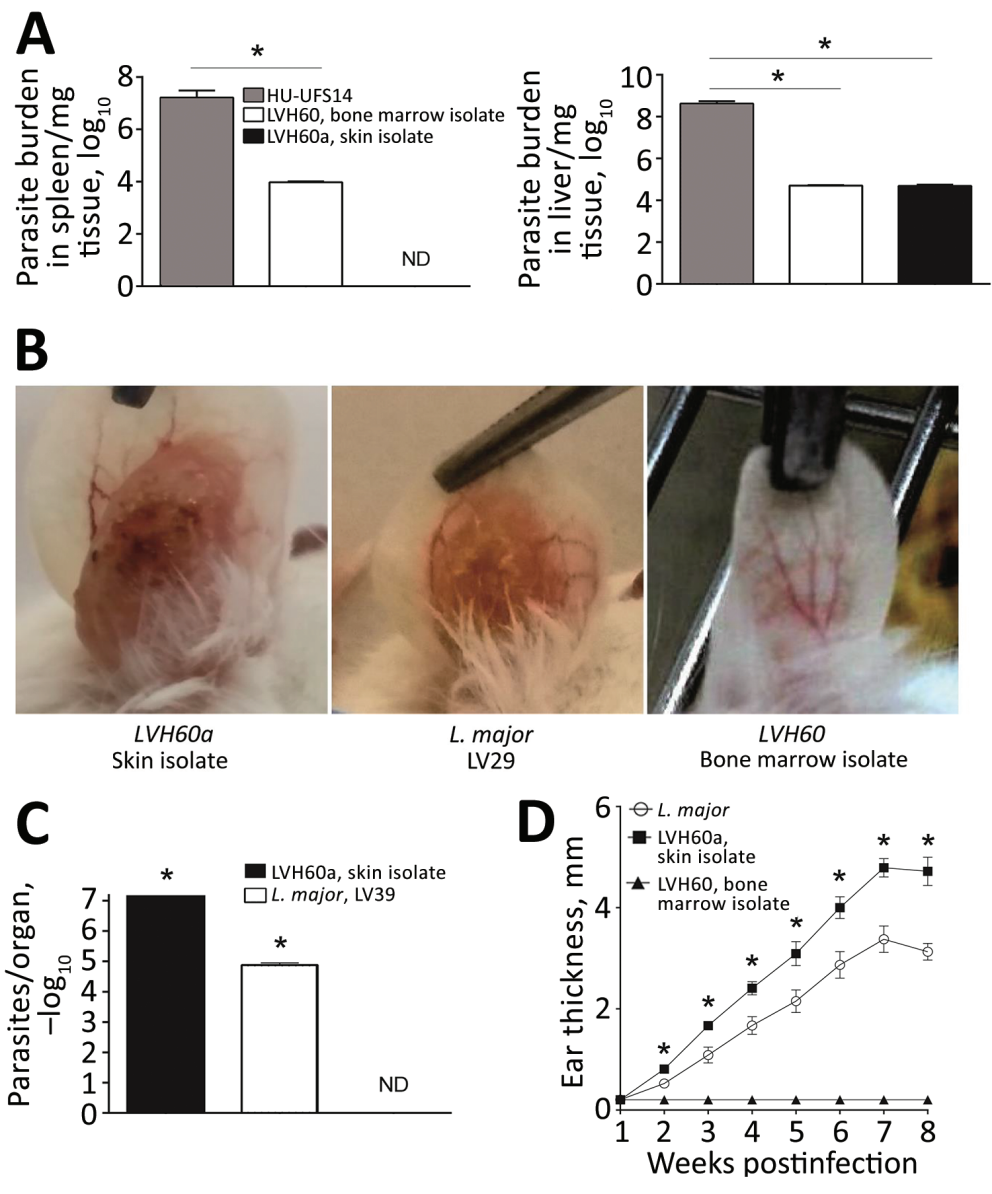
Experimental infection of BALB/c mice with LVH60 and LVH60a clinical isolates obtained from a 64-year-old man with fatal visceral leishmaniasis–like illness, Brazil. LVH60 was isolated from bone marrow, LVH60a from a skin lesion biopsy. Female BALB/c mice were infected intravenously with 10^7^ stationary-phase promastigotes. After 4 weeks of infection, spleen and liver samples were collected. Parasite loads were determined by a limiting dilution assay of spleen and liver homogenates and are expressed as the mean ± SD. A) LVH60 strain infection in mice resulted in parasite detection in the spleen and liver; the LVH60a strain was not detected in the spleen. B) For cutaneous infection, BALB/c mice were injected subcutaneously in the right ear dermis with 10^6^ stationary phase promastigotes. Infected ears were collected and imaged. C) Parasite burden in ears was assessed by a limiting dilution assay. D) Ear thickness was measured weekly with a digital caliper. The HU-UFS14 strain (*L. infantum*) was used as a positive control for experimental visceral leishmaniasis (A), whereas the LV29 strain (*L. major*) was used as a positive control for experimental cutaneous leishmaniasis. The results represent 3 independent experiments. Error bars indicate SD. ND, not detected. *p<0.05.

Because LVH60 and LVH60a were more closely related to monoxenous trypanosomatids, we performed experimental intravenous infections in BALB/c mice with these non-*Leishmania* clinical isolates or the HU-UFS14 strain to evaluate their infectious capacity. We analyzed parasite load in the spleen and liver. We found the LVH60 and LVH60a strains in the liver, although at much lower levels than HU-UFS14. However, in the spleen, we detected only LVH60 (Figure 2, panel A). Because LVH60a was isolated from the skin and both LVH60 and LVH60a were barely detected in organs, we infected BALB/c mice with these parasites through the intradermal route on the ears to evaluate their capacity to generate skin lesions and compared the results with those obtained with *L. major* LV29, the positive control.

Only the LVH60a strain was able to establish infection and cause ear lesions (Figure 2, panel B), as measured by parasite load (Figure 2, panel C) and ear thickness (Figure 1, panel D). The injury caused by LVH60a to the ear skin was more extensive than that resulting from the *L. major* LV29-positive control. Thus, the phenotypes observed with experimental infection corroborate the clinical manifestations in the patient; that is, the LVH60a strain isolated from skin lesions injured the skin tissue of mice under experimental cutaneous infection. Thus, these parasite strains closely related to *C. fasciculata* can be considered a new dixenous parasite able to infect mammals, such as humans and mice.

## Conclusions

Our study showed that non-*Leishmania*, *Crithidia*-related parasites were involved in an atypical manifestation similar to VL in this patient. Because few drugs exist with which to treat leishmaniasis, this identification of a new trypanosomatid strain refractory to treatment that can cause disease either as a single infection or as a co-infection with *Leishmania* is serious and might increase the problem of disease control. This scenario highlights the urgent need for studies of new drugs to treat this new strain. Moreover, the fact that this parasite appeared in a sister phylogenetic position to *C. fasciculata* focuses attention on potential vectors because leishmaniasis is transmitted by female sand flies, whereas *C. fasciculata* infects only anopheline and *Culex* mosquitoes. Recently, both *C. fasciculata* and *L. infantum* sequences were detected in phlebotomine *Nyssomyia whitmani* samples collected in the northern region of Brazil (*15*). Our findings raise concerns about the need to isolate and characterize parasites from more humans, reservoirs, and vectors; map trypanosomatid distribution and epidemiologic control measures; study the sensitivity of these parasites to drugs and design new treatment options; and develop new epidemiologic/ecologic strategies to control *Crithidia-*related species.

Appendix Additional methods and results for study of non-*Leishmania* parasite in fatal visceral leishmaniasis–like disease, Brazil.
