# Supplementary material for: Non-Leishmania Parasite in Fatal Visceral Leishmaniasis–Like Disease, Brazil
Source: Emerg Infect Dis. 2019 Nov;25(11):2088–92. doi: 10.3201/eid2511.181548 (PMC6810192; doi:10.3201/eid2511.181548)
Supplement: Appendix — Additional methods and results for study of non-Leishmania parasite in fatal visceral leishmaniasis–like disease, Brazil. [file 18-1548-Techapp-s1.pdf]

Article DOI: https://doi.org/10.3201/eid2511.181548

Non-*Leishmania* Parasite in Fatal Visceral Leishmaniasis–like Disease, Brazil

Appendix

1. Methods

1.1. Clinical Isolates

Three clinical isolates and 3 *Leishmania* reference strains were used in the study. The parasite strain HU-UFS14 was isolated from bone marrow aspirates of a patient with a classical clinical presentation and confirmed diagnosis of visceral leishmaniasis (VL) at the University Hospital of the Federal University of Sergipe, Aracaju, Sergipe, Brazil. This strain has been previously characterized as *L. infantum*. The parasite strains LVH60 and LVH60a were isolated from the same patient, who developed treatment-refractory VL-like disease and disseminated papular skin lesions (DPSL), without ulceration, after 8 months of treatment, resembling a diffuse cutaneous leishmaniasis. LVH60 was isolated from the bone marrow, and LVH60a was isolated from the skin papules. VL diagnosis was confirmed by light microscopic examination of amastigotes in bone marrow aspirates from bone marrow and promastigotes in culture upon parasite isolation, in addition to a positive rK39 serologic test (Kalazar Detect Rapid Test, InBios International, Seattle, Washington, USA). The HU-UFS14 strain was used as a reference *L. infantum chagasi* and has been used in several studies in a murine model of visceral leishmaniasis (*1*–*5*). *Leishmania* species identification by isoenzyme electrophoresis (*6*) for LVH60 and LVH60a isolates was inconclusive (performed at the Leishmaniasis Research Laboratory, Oswaldo Cruz Institute, Rio de Janeiro, Rio de Janeiro, Brazil). Following parasite isolation in culture medium, clinical isolates were cryopreserved after 4–6 days of culture. The WHO reference strains MHOM/BR/74/PP75 (*L. infantum*) and MHOM/BR/75/M2903 (*L. braziliensis*) were used for comparison in the morphological analysis. *L. major* LV29 was used as a control for the ear infection experiments in BALB/c mice. All procedures were performed following approval from local ethics committees (Ethics Committee of the University Hospital, Federal University of Sergipe, #CAAE 0151.0.107.000–07, #CAAE 04587312.2.0000.0058). Subjects or their legal guardians signed an informed consent form.

1.2. Parasite Cultures

Promastigotes of each clinical isolate strain (original stock) were defrosted and cultured in Schneider's insect medium (Sigma-Aldrich) with 10% heat-inactivated fetal bovine serum, 5% penicillin/streptomycin, and 2% male human urine at 25°C during 3–4 days. At the second passage, promastigote cultures were grown for 3–4 days until they reached ≈$2x10^7$ parasites/mL.

Clonal colonies were obtained by plating 40 µL of promastigote cultures of LVH60 and LVH60a clinical isolates (3 days of culture) in 1,5% bacteriological agar plates associated with complete Schneider's medium (20% FBS, 2% male urine and 5% penicillin-streptomycin-

glutamine) and kept at 26°C for 5–10 days or until clonal colonies were visible. The non-confluent colonies were selected, picked up with sterile toothpick, and then transferred to cell culture flasks containing complete Schneider's medium for the growth of clones in culture.

Cultures (both original stocks and respective clones) were washed with PBS, centrifuged and harvested as promastigote pellets used for genomic DNA extraction with a Wizard® Genomic DNA Purification Kit (Promega) following the manufacturer's instructions. DNA samples were quantified using a NanoDrop ND-1000 spectrophotometer (Thermo Scientific) and were stored at −20°C until subjected to deep sequencing and/or amplicon detection by PCR.

Smears from promastigote cultures were prepared on microscope slides and stained with a Rapid Panoptic Staining Kit (Laborclin). Light microscope images of parasites were captured with an immersion objective. Promastigotes of the WHO reference strains MHOM/BR/74/PP75 (*L. infantum*) and MHOM/BR/75/M2903 (*L. braziliensis*) were used for comparison.

1.3. PCR Detection and Whole-Genome Sequencing

PCR amplifications for species typing were performed for the i) small subunit rRNA (SSU rRNA) region with the primers TRY927F/R and SSU561F/R, as described elsewhere (*7*), ii) ribosomal internal transcribed spacer 1 (ITS1) region with the primers LITSR and L5.8S, as described elsewhere (*8*) and iii) glyceraldehyde 3-phosphate dehydrogenase gene (GAPDH) with the primers GAPTRY-modF and GAPTRYrR as described elsewhere (*9*). These amplicons have been extensively used for molecular taxonomy of trypanosomatids (*9–11*). Also, primers for heat-shock protein 70 gene (HSP70), F25 and R1310, which have been used to discriminate species of *Leishmania* (*12–15*) were used as described elsewhere (*16*). PCR products were purified using a PureLink PCR Purification kit (Invitrogen) and were sequenced using a BigDye® Terminator v3.1 Cycle Sequencing Kit (Applied Biosystems) through the dideoxy method (Sanger sequencing) using an Applied Biosystems 3130 Genetic Analyzer.

Whole-genome sequencing was performed under the Illumina protocol with 50 ng of genomic DNA from uncloned promastigote cultures (second passage), using a Nextera® XT DNA Library Preparation Kit and a HiSeq® Rapid SBS Kit v2 (500 cycles, 2x250 bp paired-end reads) on a HiSeq® 2500 Rapid Run mode system, per the manufacturer's instructions, at the ESALQ Genomics Center, University of São Paulo, Piracicaba, São Paulo, Brazil. DNA libraries were sequenced in duplicate. The same libraries were sequenced in 2 lanes of a rapid mode flow cell. An average of 47.3 million paired-end reads were obtained for each sequenced isolate. Approximately 20% of paired-end reads for each sequenced sample were discarded during the filtering step due to low quality or primer contamination.

1.4. Histopathology and DNA isolation from patient tissues

For histopathological analysis, tissue samples obtained from the skin and liver were collected and processed. The tissues were fixed in formalin, dehydrated in graded ethanol and embedded in paraffin. Serial sections (5 µm) were cut and mounted on glass slides precoated with 0.1% poly-L-lysine (Sigma-Aldrich). Histological assessment was performed after routine hematoxylin-eosin staining. The areas of the skin and liver lesions were determined using Leica Qwin software (Mannheim, Germany). For genomic DNA extraction from blood, bone marrow,

skin biopsy, liver and spleen, we used a PureLink® Genomic DNA Mini Kit (Invitrogen) according to the manufacturer's instructions.

1.5. Molecular Karyotyping of Clinical Isolates by Pulsed-Field Gel Electrophoresis (PFGE)

The clones for each isolate were selected by plating the cultures on M199 medium, supplemented with 10% heat-inactivated fetal bovine serum, 1% penicillin and streptomycin, plus noble agar (all from Sigma-Aldrich). The plates were maintained at 24°C for 10–15 days to allow the parasites to grow, as previously described (*17*). Chromosomal fingerprints for both isolate cultures and clones were assessed by PFGE as described elsewhere (*17*,*18*). Briefly, promastigote samples were immobilized in 2% low-melting temperature agarose plugs, incubated in lysis solution (0.5M EDTA pH 9.0, 1% sodium lauroyl sarcosinate, 0.5 mg/mL proteinase K) for 48 hours at 45°C and then loaded in gels running in a contour-clamped homogeneous electric field apparatus (CHEF DR II, BioRad, USA) at 14°C, at 4.5 V cm^{-1} over 48 hours with a 50- to 120-s pulse ramp time. A Lambda Ladder of size range 50–1,000 kb was used as a standard size marker for PFGE (New England Biolabs). Gels were stained with ethidium bromide (0.5 µg/mL final concentration), and electrophoresis gel images were captured with an ImageQuant LAS 4000 System.

1.6. Experimental Infections in Mice

Female BALB/c mice used for the experiments were maintained in temperature-controlled rooms (22 to 25°C) at the animal facility of the Ribeirão Preto Medical School, University of São Paulo. The mice received water and food ad libitum under pathogen-free conditions. All experimental procedures were approved by the Ethics in Animal Experimentation Committee (CETEA) from Ribeirão Preto Medical School (approval 046/2012). BALB/c mice were anesthetized using 100 mg/kg ketamine–12.5 mg/kg xylazine (administered intraperitoneally [i.p.]) and were intravenously infected with 10^7 promastigotes in 100 µL injected into the retro-orbital plexus. Four weeks post-infection, spleens and livers were collected to assess parasite titers by a quantitative limiting dilution assay as previously described (*19*,*20*). For cutaneous infections, mice were infected subcutaneously with stationary phase promastigotes (10^6 parasites in 10 µL of sterile PBS) in the right ear dermis using a 27.5-gauge needle. Lesion size was defined as the difference in thickness between the infected ear and the uninfected contralateral ear. Disease progression was monitored weekly using a digital caliper (Mitutoyo, Suzano, SP, Brazil), and the parasite load was determined using a limiting dilution assay.

1.7. Bioinformatics Analysis

Quality control of raw sequence data was performed with FastQC (http://www.bioinformatics.babraham.ac.uk/projects/fastqc/). Sequencing adaptors/primers and low-quality reads were trimmed used Trim Galore version 0.4.1 (https://www.bioinformatics.babraham.ac.uk/projects/trim_galore/), yielding 96–186 million reads per sequenced parasite (Appendix Table 2). High-quality reads were used to assemble contigs using Abyss (*21*) with a k-mer size of 96, generating genomes varying from 32–54 Mb (Appendix Table 3). The choice of k-mer size was based on a previous test, in which a range of k-mer values starting from 26 to 96 varying every 10 (26, 36, 46…96) were used to assemble the reads. The k-mer size of 96 yielded the less fragmented assembly.

Coding sequences (CDSs) from all 6 open reading frames (ORFs) were extracted from contig consensus sequences using the EMBOSS (*22*) tool. Translated CDSs were validated and adjusted to the starting methionine through BLAST-P (*23*) searches against protein sequences from the NCBI RefSeq (*24*) Protozoa and UniProtKB/Swiss-Prot (*25*) databases. Annotated protein sequences of kinetoplastid reference genomes were retrieved from NCBI or other sites (Appendix Table 4). When protein sequences were unavailable for publicly available genomes, CDSs were extracted following the same protocol as that used for clinical isolate sequences.

Orthologous identification among reference genomes and deduced protein sequences from clinical isolates was performed with the reciprocal smallest distance (RSD) algorithm (*26*). A distance matrix was computed using the median amino acid distance of orthologous peptidesequence pairs found in all organism pairs (Appendix Tables 4, 5). A hierarchical clustering dendrogram of median values from orthologous distance matrices was constructed with the pvclust (*27*) R package using the Euclidean distance method.

Phylogenetic analysis was performed with nucleotide (SSU rRNA and ITS1) and protein (GAPDH, glyceraldehyde 3-phosphate dehydrogenase) sequences of Trypanosomatidae. Coding sequences for GAPDH were translated in the 3 clinical isolates and compared to respective orthologous proteins from other trypanosomatid organisms (accession numbers are available in Appendix Table 10). Nucleotide sequences for the SSU rRNA sequences were retrieved from NCBI based on the BLAST-N results. Phylogenetic trees were constructed with the maximum-likelihood (ML) method with a Jones-Taylor-Thornton (JTT) matrix-based substitution model (*28*) for protein sequences and a Tamura-Nei substitution model for nucleotide sequences. Bootstrapping tests were performed with 1,000 replicates. All multiple sequence alignments and phylogenies were generated using MEGA 6 (*29*) software.

1.8. Data Availability

The sequences and assemblies generated in this work are registered with GenBank/NCBI under BioProject accession number PRJNA398352. All related accession numbers are available in Appendix Tables 7 and 9).

2. Orthologous Protein Sequences

Orthologous protein sequences of trypanosomatids species analyzed in this work are available at http://exon.niaid.nih.gov/transcriptome/Cridia/fasta.zip by request.

Appendix Table 1. Percent nucleotide identity matrix for ribosomal internal transcribed spacer 1 (ITS1*) sequences from LVH60 and LVH60a clinical isolates (and their respective clones) compared to *Leishmania* and *Crithidia* species*

Species	*C. luciliae*	LVH60a clo.1	LVH60a clo.2	LVH60 clo.6	LVH60 clo.1	LVH60	LVH60a	*C. fasciculata*	*L. braziliensis*	*L. panamensis*	*L. guyanensis*	*L. mexicana*	*L. amazonensis*	*L. aethiopica*	*L. tropica*	*L. donovani*	*L. infantum*	*L. chagasi*	*L. major*	*L. gerbilli*
C. luciliae	100.0	91.3	92.1	92.1	92.1	92.1	92.1	92.1	46.8	46.8	46.8	44.7	44.7	47.0	48.9	49.0	48.8	48.2	46.5	45.4
LVH60a clo.1	91.3	100.0	98.9	98.4	98.9	98.7	98.9	98.9	52.8	52.8	52.8	49.8	49.8	52.3	54.4	54.0	54.3	54.8	51.8	50.9
LVH60a clo.2	92.1	98.9	100.0	99.2	100.0	99.5	100.0	100.0	53.6	53.6	53.6	50.6	50.6	53.0	55.1	54.8	54.7	55.1	52.1	51.3
LVH60 clo.6	92.1	98.4	99.2	100.0	99.2	99.7	100.0	99.7	53.6	53.6	53.6	50.6	50.6	53.0	55.1	54.8	54.7	54.2	52.1	51.3
LVH60 clo.1	92.1	98.9	100.0	99.2	100.0	99.5	100.0	100.0	53.6	53.6	53.6	50.6	50.6	53.0	55.1	54.8	54.7	55.1	52.1	51.3
LVH60	92.1	98.7	99.5	99.7	99.5	100.0	100.0	100.0	53.6	53.6	53.6	50.6	50.6	53.0	55.1	54.8	54.7	54.6	52.1	51.3
LVH60a	92.1	98.9	100.0	100.0	100.0	100.0	100.0	100.0	53.6	53.6	53.6	50.6	50.6	53.0	55.1	54.8	54.7	54.8	52.1	51.3
C. fasciculata	92.1	98.9	100.0	99.7	100.0	100.0	100.0	100.0	53.6	53.6	53.6	50.6	50.6	53.0	55.1	54.8	54.7	55.0	52.1	51.3
L. braziliensis	46.8	52.8	53.6	53.6	53.6	53.6	53.6	53.6	100.0	99.2	99.2	65.1	64.1	68.8	70.5	69.9	70.2	70.2	65.4	67.1
L. panamensis	46.8	52.8	53.6	53.6	53.6	53.6	53.6	53.6	99.2	100.0	100.0	64.8	63.8	68.8	70.5	69.9	70.2	70.2	65.4	67.1
L. guyanensis	46.8	52.8	53.6	53.6	53.6	53.6	53.6	53.6	99.2	100.0	100.0	64.8	63.8	68.8	70.5	69.9	70.2	70.2	65.4	67.1
L. mexicana	44.7	49.8	50.6	50.6	50.6	50.6	50.6	50.6	65.1	64.8	64.8	100.0	97.3	78.4	80.9	80.2	79.8	77.7	69.9	71.3
L. amazonensis	44.7	49.8	50.6	50.6	50.6	50.6	50.6	50.6	64.1	63.8	63.8	97.3	100.0	79.9	80.2	80.7	79.9	78.5	70.0	71.1
L. aethiopica	47.0	52.3	53.0	53.0	53.0	53.0	53.0	53.0	68.8	68.8	68.8	78.4	79.9	100.0	87.5	79.5	79.1	77.7	80.6	79.7
L. tropica	48.9	54.4	55.1	55.1	55.1	55.1	55.1	55.1	70.5	70.5	70.5	80.9	80.2	87.5	100.0	86.3	86.4	84.8	80.6	83.0
L. donovani	49.0	54.0	54.8	54.8	54.8	54.8	54.8	54.8	69.9	69.9	69.9	80.2	80.7	79.5	86.3	100.0	97.1	96.8	87.7	85.9
L. infantum	48.8	54.3	54.7	54.7	54.7	54.7	54.7	54.7	70.2	70.2	70.2	79.8	79.9	79.1	86.4	97.1	100.0	100.0	86.4	88.3
L. chagasi	48.2	54.8	55.1	54.2	55.1	54.6	54.8	55.0	70.2	70.2	70.2	77.7	78.5	77.7	84.8	96.8	100.0	100.0	86.0	88.3
L. major	46.5	51.8	52.1	52.1	52.1	52.1	52.1	52.1	65.4	65.4	65.4	69.9	70.0	80.6	80.6	87.7	86.4	86.0	100.0	92.7
L. gerbilli	45.4	50.9	51.3	51.3	51.3	51.3	51.3	51.3	67.1	67.1	67.1	71.3	71.1	79.7	83.0	85.9	88.3	88.3	92.7	100.0

*The Matrix was created by Clustal2.1 Multiple Sequence Alignment tool available at https://www.ebi.ac.uk/Tools/msa/clustalo/. A total of 399 positions were considered in the final alignment. Reference accession numbers retrieved from NCBI for: *Crithidia luciliae*: AJ627018.1; *Crithidia fasciculata:* HM004585.1; *Leishmania braziliensis*: HG512930.1; *Leishmania panamensis*: HG512959.1; *Leishmania guyanensis:* HG512915.1; *Leishmania mexicana:* HG512912.1; *Leishmania amazonensis:* HG512933.1; *Leishmania aethiopica:* HG512946.1; *Leishmania tropica*: HG512927.1; *Leishmania donovani:* MH450081.1; *Leishmania infantum:* HG512955.1; *Leishmania chagasi:* AJ000305.1; *Leishmania major*: HG512924.1; *Leishmania gerbilli:* HG512948.1. Clo#: clonal colonies for their respective parasitic strains. * Sequences were deposited in European Nucleotide Archive (ENA) under accession number study PRJEB33749 and are available at http://www.ebi.ac.uk/ena/data/view/PRJEB33749.

Appendix Table 2. Reads used for genome assembly after trimming low quality (Phred score <30) and primer sequences*

Library name (clinical isolate)	Total no. paired-end reads	Total no. nt	Average length of read, nt	Median size of read, nt	L50, nt	Coverage†
HU-UFS14 (VL)	49,914,669	20,864,018,449	209	248	244	652
LVH60 (VL-like)	93,060,789	40,069,440,382	215	248	247	1252
LVH60a (DPSL VL-like)	48,685,595	20,951,743,131	215	248	247	655

*DPSL, disseminated popular skin lesions; VL, visceral leishmaniasis.
†Based on haploid genome size of 32 Mb (*Leishmania spp*).

Appendix Table 3. Assembly specifications of clinical isolates for scaffolds >499 nt and genome specifications for other kinetoplastids deposited at NCBI*

	Brazilian clinical isolates from patients diagnosed with leishmaniasis					
Specification	No. scaffolds	Average length, nt	Median size, nt	L50, nt	Larger scaffold size, nt	Predicted haploid genome size, nt
Library name (clinical isolate)						
HU-UFS14 (VL)	2,182	14,888	30,043	7,930	182,547	32,486,338
LVH60 (VL-like)	4,522	11,913	24,329	5,472	416,301	53,870,039
LVH60a (DPSL VL-like)	4,495	12,131	23,223	5,784	410,207	54,530,145
	No. scaffolds	Average length, nt	Median size, nt	L50, nt	Larger sequence size, nt	Haploid genome size, nt
Species name						
Angomonas deanei (MXE)	408	47,180	200,001	1,800	956,813	19,249,610
Angomonas desouzai (MXE)	7,953	2,969	5,841	1,465	64,044	23,614,611
Bodo saltans (FL)	2,256	17,590	31,827	10,625	190,847	39,683,914
Crithidia acanthocephali (MX)	5,199	6,418	19,941	1,908	186,292	33,367,252
Crithidia bombi (MX)	2,896	10,838	23,620	5,053	136,502	31,385,876
Crithidia fasciculata (MX)	458	90,089	19,941	18,084	2,960,310	41,260,738
Endotrypanum monterogeii (DX)	989	32558	23620	1010	3727717	32,200,132
Herpetomonas muscarum (MX)	10,264	2,925	6,864	12,22	68,920	30,023,690
Leishmania aethiopica (DX)	159	198,934	23,620	3,567	2,678,989	31,630,583
Leishmania amazonensis (DX)	2,627	10,970	22,916	5,327	171,240	28,819,188
Leishmania braziliensis (DX)	138	232,302	22,916	7,583	2,686,563	32,057,731
Leishmania donovani (DX)	36	901,169	22,916	200,001	2,713,168	32,442,088
Leishmania enrietti (DX)	495	62,065	5,841	2,354	2,596,630	30,722,261
Leishmania gerbilli (DX)	492	63,738	200,001	4,529	1,220,768	31,359,288
Leishmania infantum (DX)	76	422,579	22,916	16,937	2,673,876	32,115,981
Leishmania major (DX)	36	912,561	6,864	200,001	2,682,071	32,852,209
Leishmania mexicana (DX)	588	54,527	22,916	1,321	3,343,418	32,061,701
Leishmania panamensis (DX)	35	876,743	22,916	200,001	2,610,089	30,685,994
Leishmania peruviana (DX)	37	889,319	200,001	200,001	2,746,426	32,904,821
Leishmania tropica (DX)	448	73,556	200,001	8,886	1,354,451	32,953,174
Leishmania turanica (DX)	336	96,110	200,001	6,443	1,294,291	32,293,127
Lotmaria passim (MX)	2,801	11,635	32,501	3,816	224,531	32,588,904
Perkinsela spp (EN)	693	13,676	57,461	2,267	853,862	9,477,801
Phytomonas spp (DX)	138	128,767	200,001	11,152	1,675,790	17,769,829
Strigomonas culicis (MXE)	3,002	7,777	23,570	2,290	158,114	23,346,993
Strigomonas galati (MXE)	7,280	3,661	6,773	1,952	55,701	26,652,600
Strigomonas oncopelti (MXE)	8,626	2,814	4,581	1,735	37,314	24,272,838
Trypanosoma brucei (DX)	11	2,013,383	4,581	200,001	4,531,529	22,147,208
Trypanosoma cruzi (DX)	29,495	2,969	95,319	863	990,640	87,577,856
Trypanosoma equiperdum (DX)	2,026	12,946	38,149	4,257	367,571	26,228,029
Trypanosoma grayi (DX)	2,871	7,212	16,882	3,286	126,695	20,704,452
Trypanosoma rangeli (DX)	9,066	1,587	2,068	1,302	25,208	14,388,992

*CL, cutaneous leishmaniasis; DX, dixenous (2-hosts) life cycle infecting both vertebrates and invertebrates; EN, obligate endosymbiont; FL, free-living nonparasitic; MCL, muco-cutaneous leishmaniasis; MX, monoxenous (1-host) life cycle only in invertebrates; MXE: endosymbiont-bearing monoxenous; PKDL, post–kala-azar dermal leishmaniasis.

Appendix Table 4. Source of Trypanosomatida genomes used in this study

Abbreviation	Species name	URL	Genome sequence source	Protein sequence source	Publication or publication date
ANGDEA	*Angomonas deanei*	https://www.ncbi.nlm.nih.gov/genome/14191	NCBI	Orf/blastx REFSEQ	31/07/13
ANGDES	*Angomonas desouzai*	https://www.ncbi.nlm.nih.gov/genome/17778	NCBI	Orf/blastx REFSEQ	25/10/13
BODSAL	*Bodo saltans*	https://www.ncbi.nlm.nih.gov/genome/41729	NCBI	NCBI	https://www.ncbi.nlm.nih.gov/pubmed/26725202
CRIACA	*Crithidia acanthocephali*	https://www.ncbi.nlm.nih.gov/genome/24422	NCBI	Orf/blastx REFSEQ	https://www.ncbi.nlm.nih.gov/pubmed/24015778
CRIBOM	*Crithidia bombi*	https://www.ncbi.nlm.nih.gov/genome/55882	NCBI	Orf/blastx REFSEQ	11/07/17
CRIFAS	*Crithidia fasciculata*	https://www.ncbi.nlm.nih.gov/genome/14517	NCBI	Orf/blastx REFSEQ	https://www.ncbi.nlm.nih.gov/pubmed/28210761

Abbreviation	Species name	URL	Genome sequence source	Protein sequence source	Publication or publication date
CRIMEL	*Crithidia mellificae*	https://www.ncbi.nlm.nih.gov/genome/11456	NCBI	Orf/blastx REFSEQ	https://www.ncbi.nlm.nih.gov/pubmed/24743507
ENDMOT	*Endotrypanum monterogeii*	https://www.ncbi.nlm.nih.gov/genome/13990	NCBI	Orf/blastx REFSEQ	18/01/13
HERMUS	*Herpetomonas muscarum*	https://www.ncbi.nlm.nih.gov/genome/24421	NCBI	Orf/blastx REFSEQ	https://www.ncbi.nlm.nih.gov/pubmed/24015778
HU-UFS14	*Leishmania infantum*	current work	current work	Orf/blastx REFSEQ	
LEIAMA	*Leishmania amazonensis*	http://bioinfo08.ibi.unicamp.br/leishmania/	http://bioinfo08.ibi.unicamp.br/leishmania/	http://bioinfo08.ibi.unicamp.br/leishmania/	https://www.ncbi.nlm.nih.gov/pubmed/23857904
LEIBRA	*Leishmania brasiliensis*	https://www.ncbi.nlm.nih.gov/genome/718	NCBI	NCBI	https://www.ncbi.nlm.nih.gov/pubmed/22038252
LEIDON	*Leishmania donovani*	https://www.ncbi.nlm.nih.gov/genome/3516	NCBI	NCBI	https://www.ncbi.nlm.nih.gov/pubmed/22038251
LEIENR	*Leishmania enrietti*	https://www.ncbi.nlm.nih.gov/genome/16917	NCBI	Orf/blastx REFSEQ	12/06/13
LEIGER	*Leishmania gerbilli*	https://www.ncbi.nlm.nih.gov/genome/30027	NCBI	Orf/blastx REFSEQ	02/08/13
LEIINF	*Leishmania infantum*	https://www.ncbi.nlm.nih.gov/genome/249	NCBI	NCBI	https://www.ncbi.nlm.nih.gov/pubmed/17572675
LEIMAJ	*Leishmania major*	https://www.ncbi.nlm.nih.gov/genome/23	NCBI	NCBI	https://www.ncbi.nlm.nih.gov/pubmed/22038252
LEIMEX	*Leishmania mexicana*	https://www.ncbi.nlm.nih.gov/genome/14469	NCBI	NCBI	https://www.ncbi.nlm.nih.gov/pubmed/22038252
LEIPAN	*Leishmania panamensis*	https://www.ncbi.nlm.nih.gov/genome/13991	NCBI	NCBI	https://www.ncbi.nlm.nih.gov/pubmed/25707621
LEIPER	*Leishmania peruviana*	https://www.ncbi.nlm.nih.gov/genome/40809	NCBI	Orf/blastx REFSEQ	17/09/15
LEITRO	*Leishmania tropica*	https://www.ncbi.nlm.nih.gov/genome/14404	NCBI	Orf/blastx REFSEQ	11/06/13
LEITUR	*Leishmania turanica*	https://www.ncbi.nlm.nih.gov/genome/30020	NCBI	Orf/blastx REFSEQ	29/07/13
LEPPYR	*Leptomonas pyrrhocoris*	https://www.ncbi.nlm.nih.gov/genome/40172	NCBI	NCBI	https://www.ncbi.nlm.nih.gov/pubmed/27021793
LEIEAT	*Leishmania aethiopica*	https://www.ncbi.nlm.nih.gov/genome/14335	NCBI	Orf/blastx REFSEQ	05/08/13
LOTPAS	*Lotmaria passim*	https://www.ncbi.nlm.nih.gov/genome/36572	NCBI	NCBI	https://www.ncbi.nlm.nih.gov/pubmed/24743507
LVH60	*New species*	current work	current work	Orf/blastx REFSEQ	
LVH60a	*New species*	current work	current work	Orf/blastx REFSEQ	
PERSPE	*Perkinsela sp.*	https://www.ncbi.nlm.nih.gov/genome/33979	NCBI	NCBI	https://www.ncbi.nlm.nih.gov/pubmed/26628723
PHYSPE	*Phytomonas sp.*	https://www.ncbi.nlm.nih.gov/genome/14536	NCBI	NCBI	https://www.ncbi.nlm.nih.gov/pubmed/24516393
STRCUL	*Strigomonas culicis*	https://www.ncbi.nlm.nih.gov/genome/24420	NCBI	Orf/blastx REFSEQ	31/07/13
STRGAL	*Strigomonas galati*	https://www.ncbi.nlm.nih.gov/genome/24419	NCBI	Orf/blastx REFSEQ	25/10/13
STRONC	*Strigomonas oncopelti*	https://www.ncbi.nlm.nih.gov/genome/24	NCBI	NCBI	https://www.ncbi.nlm.nih.gov/pubmed/20404998
TRYBRU	*Trypanosoma brucei*	https://www.ncbi.nlm.nih.gov/genome/10876	NCBI	NCBI	https://www.ncbi.nlm.nih.gov/pubmed/25233456
TRYCRU	*Trypanosoma congolense*	https://www.ncbi.nlm.nih.gov/genome/25	NCBI	NCBI	https://www.ncbi.nlm.nih.gov/pubmed/22331916
TRYEQU	*Trypanosoma cruzi*	https://www.ncbi.nlm.nih.gov/genome/41709	NCBI	NCBI	https://www.ncbi.nlm.nih.gov/pubmed/24482508
TRYGRA	*Trypanosoma equiperdum*	https://www.ncbi.nlm.nih.gov/genome/31973	NCBI	NCBI	https://www.ncbi.nlm.nih.gov/pubmed/28138343
TRYRAN	*Trypanosoma grayi*	https://www.ncbi.nlm.nih.gov/genome/10993	NCBI	NCBI	https://www.ncbi.nlm.nih.gov/pubmed/25977781

Appendix Table 5. Number of orthologous pairs determined by paired comparisons through the reciprocal smallest distance algorithm (RSD), with a minimum of 1,357 protein comparisons in 1 comparison pair, and an average of 6,387 proteins per compared pair. Related to Figure 2. Protein sequences of orthologous genes were compiled in a compressed .zip file and are available at http://exon.niaid.nih.gov/transcriptome/Cridia/fasta.zip by request.

Abbrev	ANGDEA	ANGDES	BODSAL	CRIACA	CRIBOM	CRIFAS	ENDMOT	HERMUS	HU-UFS14	LEIAMA	LEIEAE	LEIENR	LEIGER	LEIBRA	LEIDON	LEIINF	LEIMAJ	LEIMEX	LEIPAN	LEIPER	LEPPYR	LEITRO	LEITUR	LOTPAS	LVH60	LVH60a	PERSPE	PHYSPE	STRGAL	STRCUL	STRONC	TRYBRU	TRYCRU	TRYEQU	TRYGRA	TRYRAN
ANGDEA	5977	4629	4289	5065	4971	5212	5012	4949	5039	4883	4990	4974	5059	4956	4981	5022	5024	5032	4972	4422	5240	5033	5054	5143	5088	5091	1665	3964	5017	5009	4943	4515	4826	4328	4855	4066
ANGDES	4765	6240	3996	4787	4664	4897	4706	4675	4702	4530	4662	4654	4727	4647	4653	4695	4698	4705	4650	4190	4901	4698	4732	4827	4779	4776	1609	3754	4776	4754	4706	4239	4529	4044	4539	3802
BODSAL	4446	4023	17840	5230	4959	5532	5180	5044	5335	5102	5244	5182	5271	5226	5256	5317	5323	5312	5249	4494	5475	5266	5310	5358	5348	5358	1864	4170	4657	4669	4565	5198	5881	4970	5862	4760
CRIACA	5269	4814	5227	11800	6872	8064	6950	6203	7177	6794	7148	6967	7202	7025	7075	7179	7150	7151	7026	6095	8000	7187	7208	7801	7498	7506	1826	4991	5573	5581	5483	5706	6269	5446	6324	5106
CRIBOM	5160	4697	4969	6901	7675	7109	6421	5841	6568	6300	6536	6417	6610	6421	6464	6551	6540	6537	6431	5690	7093	6576	6614	6936	6756	6767	1818	4713	5422	5447	5358	5384	5847	5139	5891	4843
CRIFAS	5572	5075	5679	8258	7267	13212	7381	6545	7716	7286	7601	7403	7661	7570	7607	7719	7705	7692	7562	6442	8576	7657	7682	8196	8422	8449	1982	5357	5866	5882	5769	6171	6795	5880	6766	5513
ENDMOT	5190	4716	5154	6920	6392	7172	8134	6001	7186	6836	7125	7013	7199	7047	7080	7163	7156	7155	7056	6060	7228	7164	7213	7061	7056	7060	1849	5089	5455	5491	5357	5811	6206	5535	6222	5128
HERMUS	5139	4697	5043	6199	5834	6369	6014	10297	6122	5859	6071	6002	6143	6001	6040	6102	6095	6104	6012	5264	6380	6117	6150	6307	6188	6189	1802	4755	5431	5438	5327	5376	5808	5132	5873	4806
HU-UFS14	5269	4802	5402	7241	6623	7614	7275	6194	8148	7303	7592	7313	7657	7544	7842	7957	7791	7752	7549	6338	7619	7634	7665	7383	7527	7540	1951	5226	5578	5589	5489	5995	6471	5708	6448	5368
LEIAMA	5067	4571	5115	6805	6299	7127	6862	5873	7257	7299	7095	6893	7156	7065	7143	7226	7209	7254	7078	5991	7158	7136	7164	6959	7069	7079	1824	4976	5306	5334	5237	5702	6133	5448	6134	5069
LEIEAE	5137	4655	5209	7077	6472	7359	7099	6014	7462	7046	8290	7189	7593	7209	7355	7427	7419	7387	7217	6201	7395	7625	7602	7225	7190	7194	1810	5036	5429	5428	5326	5770	6240	5509	6263	5133
LEIENR	5127	4659	5157	6932	6389	7188	7002	5970	7226	6863	7216	7999	7279	7102	7116	7199	7191	7183	7112	6121	7227	7253	7281	7069	7037	7047	1831	5028	5388	5408	5293	5743	6193	5491	6215	5103
LEIGER	5226	4731	5248	7153	6557	7435	7185	6111	7542	7116	7611	7266	8399	7298	7435	7502	7517	7471	7303	6279	7458	7658	7770	7294	7271	7284	1839	5101	5500	5513	5411	5870	6308	5604	6338	5183
LEIBRA	5199	4743	5283	7091	6483	7460	7140	6061	7543	7102	7336	7199	7410	8151	7459	7576	7560	7556	7643	6502	7481	7378	7422	7242	7377	7383	1910	5178	5485	5507	5392	5902	6397	5624	6335	5252
LEIDON	5197	4712	5287	7105	6489	7462	7140	6073	7795	7174	7478	7190	7533	7426	7957	7819	7673	7647	7422	6228	7489	7529	7556	7258	7380	7387	1882	5129	5450	5482	5374	5867	6354	5589	6315	5223
LEIINF	5282	4815	5403	7273	6632	7636	7286	6186	7942	7278	7598	7313	7641	7599	7876	8141	7867	7829	7570	6349	7656	7626	7655	7395	7541	7548	1948	5225	5578	5598	5507	5973	6480	5684	6443	5348
LEIMAJ	5305	4850	5429	7288	6664	7646	7313	6220	7885	7311	7631	7350	7695	7639	7796	7928	8306	7874	7612	6383	7680	7647	7700	7404	7571	7573	1956	5252	5595	5622	5509	6041	6502	5723	6473	5364
LEIMEX	5282	4822	5389	7248	6626	7606	7273	6189	7780	7321	7547	7303	7615	7589	7714	7838	7830	8137	7569	6341	7641	7579	7628	7376	7527	7539	1937	5231	5576	5604	5472	6008	6476	5698	6455	5343
LEIPAN	5192	4717	5280	7065	6468	7415	7123	6045	7517	7098	7327	7186	7399	7609	7420	7516	7510	7512	7742	6374	7450	7360	7410	7205	7336	7340	1909	5166	5464	5492	5370	5902	6378	5624	6317	5243
LEIPER	3720	3471	3598	4791	4564	4913	4763	4216	4918	4741	4891	4814	4944	4993	4829	4893	4889	4885	4960	5505	4941	4944	4955	4865	4826	4825	1357	3483	3934	3943	3864	3907	4196	3741	4248	3474
LEPPYR	5463	4938	5492	8022	7095	8413	7271	6390	7574	7159	7480	7273	7522	7419	7470	7577	7562	7558	7419	6314	9284	7513	7542	8038	7805	7813	1905	5239	5721	5732	5625	6000	6595	5719	6598	5374
LEITRO	5167	4678	5223	7096	6497	7392	7110	6053	7485	7084	7616	7209	7627	7223	7394	7452	7446	7410	7234	6230	7405	8376	7637	7226	7218	7224	1819	5049	5456	5464	5350	5814	6286	5551	6311	5154
LEITUR	5203	4720	5274	7135	6556	7418	7180	6103	7524	7112	7598	7250	7744	7280	7427	7488	7489	7459	7288	6263	7440	7640	8306	7297	7265	7273	1840	5105	5495	5511	5388	5855	6306	5598	6342	5184
LOTPAS	5357	4882	5395	7826	6956	8058	7108	6323	7348	6966	7308	7125	7362	7197	7244	7328	7302	7303	7184	6206	8053	7341	7384	12838	7537	7546	1873	5157	5684	5669	5572	5914	6428	5602	6523	5274
LVH60	5516	5066	5675	7833	7044	8659	7363	6476	7763	7361	7549	7354	7616	7619	7665	7769	7755	7750	7605	6462	8126	7595	7626	7822	9982	9753	2037	5437	5809	5834	5721	6236	6772	5930	6711	5584
LVH60a	5525	5068	5670	7824	7054	8678	7366	6464	7779	7367	7552	7367	7634	7641	7677	7783	7771	7764	7621	6466	8134	7605	7648	7825	9771	10046	2025	5452	5809	5839	5720	6225	6784	5935	6721	5590
PERSPE	1722	1624	1865	1826	1811	1880	1860	1803	1882	1809	1821	1838	1845	1863	1867	1876	1867	1866	1881	1689	1902	1833	1853	1865	1881	1870	4850	1661	1788	1793	1775	1915	1933	1847	1870	1709
PHYSPE	4118	3780	4167	4990	4708	5202	5109	4755	5164	4963	5068	5055	5136	5120	5093	5152	5157	5155	5138	4393	5231	5104	5149	5121	5123	5135	1661	6410	4315	4342	4246	4823	5050	4608	4954	4240
STRGAL	5206	4803	4651	5568	5401	5698	5462	5424	5502	5286	5463	5412	5524	5413	5420	5482	5479	5484	5421	4837	5707	5506	5535	5652	5549	5556	1785	4310	7309	5785	5880	4917	5277	4701	5303	4408
STRCUL	5205	4774	4666	5574	5440	5721	5512	5435	5525	5320	5468	5431	5543	5445	5451	5511	5511	5517	5456	4855	5720	5516	5555	5647	5589	5600	1795	4335	5787	6777	5690	4929	5293	4704	5328	4416
STRONC	5124	4729	4553	5469	5340	5589	5366	5316	5411	5216	5352	5313	5428	5321	5341	5407	5395	5379	5324	4752	5603	5396	5427	5542	5472	5473	1770	4233	5874	5685	6754	4833	5171	4619	5206	4328
TRYBRU	4687	4286	5209	5725	5395	6023	5845	5394	5933	5698	5829	5782	5919	5861	5848	5903	5943	5940	5882	4983	6009	5885	5923	5893	5944	5935	1928	4838	4938	4949	4864	9642	6582	6729	6510	5406
TRYCRU	5045	4599	5948	6327	5902	6691	6281	5855	6457	6166	6338	6276	6404	6396	6391	6450	6440	6445	6400	5395	6641	6402	6413	6424	6492	6496	1983	5129	5335	5349	5236	6620	19062	6255	7591	6334
TRYEQU	4469	4066	4963	5443	5136	5711	5552	5129	5636	5434	5548	5513	5630	5574	5549	5611	5621	5626	5594	4752	5712	5592	5639	5567	5640	5642	1844	4603	4704	4706	4629	6703	6198	7659	6169	5138
TRYGRA	5038	4564	5854	6319	5883	6570	6248	5870	6366	6119	6317	6242	6378	6264	6273	6349	6344	6362	6280	5368	6585	6370	6394	6468	6358	6367	1864	4950	5306	5329	5215	6482	7472	6170	10576	5907
TRYRAN	4215	3834	4767	5115	4833	5375	5157	4821	5290	5064	5174	5137	5224	5209	5200	5267	5250	5265	5218	4415	5363	5216	5239	5245	5269	5271	1717	4258	4421	4427	4343	5400	6263	5156	5937	7330
Minimum	1357																																			

Appendix Table 6. Calculated median values for orthologous distance matrix used for cladogram analysis. Related to Figure 2.

Abbrev	ANGDEA	ANGDES	BODSAL	CRIACA	CRIBOM	CRIFAS	ENDMOT	HERMUS	HU-UFS14	LEIAMA	LEIEAE	LEIENR	LEIGER	LEIBRA	LEIDON	LEIINF	LEIMAJ	LEIMEX	LEIPAN	LEIPER	LEPPYR	LEITRO	LEITUR	LOTPAS	LVH60	LVH60a	PERSPE	PHYSPE	STRGAL	STRCUL	STRONC	TRYBRU	TRYCRU	TRYEQU	TRYGRA	TRYRAN
ANGDEA	0.000	0.461	1.516	1.061	1.053	1.050	1.110	1.048	1.075	1.078	1.091	1.092	1.089	1.090	1.066	1.071	1.078	1.073	1.096	1.052	1.079	1.083	1.085	1.067	1.052	1.050	2.047	1.152	0.835	0.858	0.845	1.309	1.201	1.301	1.195	1.205
ANGDES	0.462	0.000	1.454	1.008	1.002	1.005	1.056	0.999	1.029	1.031	1.042	1.049	1.040	1.034	1.022	1.025	1.029	1.026	1.039	1.014	1.026	1.037	1.038	1.015	1.006	1.005	1.989	1.128	0.800	0.815	0.807	1.257	1.161	1.255	1.148	1.163
BODSAL	1.523	1.453	0.000	1.555	1.510	1.548	1.570	1.497	1.544	1.552	1.567	1.548	1.557	1.555	1.534	1.543	1.552	1.546	1.553	1.494	1.565	1.563	1.562	1.562	1.543	1.543	2.017	1.584	1.480	1.470	1.467	1.549	1.501	1.548	1.492	1.478
CRIACA	1.066	1.006	1.556	0.000	0.275	0.344	0.648	1.008	0.551	0.552	0.571	0.586	0.564	0.577	0.546	0.549	0.555	0.555	0.578	0.545	0.331	0.565	0.563	0.318	0.333	0.332	2.054	1.167	1.002	0.990	1.002	1.330	1.229	1.326	1.211	1.198
CRIBOM	1.054	1.000	1.508	0.274	0.000	0.335	0.610	0.988	0.518	0.526	0.533	0.557	0.530	0.542	0.514	0.517	0.523	0.525	0.542	0.525	0.309	0.529	0.530	0.304	0.329	0.329	2.024	1.127	0.991	0.987	1.005	1.289	1.173	1.277	1.160	1.157
CRIFAS	1.035	0.987	1.518	0.337	0.328	0.000	0.640	0.978	0.553	0.554	0.566	0.577	0.559	0.574	0.549	0.550	0.555	0.552	0.573	0.543	0.372	0.561	0.562	0.365	0.012	0.012	1.977	1.146	0.978	0.970	0.969	1.304	1.210	1.300	1.184	1.166
ENDMOT	1.111	1.055	1.572	0.645	0.610	0.653	0.000	1.074	0.408	0.410	0.417	0.451	0.416	0.430	0.402	0.406	0.410	0.411	0.432	0.411	0.657	0.411	0.415	0.658	0.653	0.653	2.037	1.194	1.064	1.051	1.068	1.367	1.249	1.360	1.238	1.231
HERMUS	1.052	0.999	1.497	1.008	0.989	0.996	1.075	0.000	1.025	1.017	1.046	1.038	1.034	1.041	1.013	1.017	1.021	1.023	1.042	0.994	1.025	1.035	1.039	1.023	1.004	1.004	2.030	1.048	0.999	0.987	0.996	1.256	1.149	1.248	1.125	1.123
HU-UFS14	1.065	1.011	1.530	0.545	0.514	0.558	0.404	1.011	0.000	0.087	0.054	0.269	0.058	0.240	0.004	0.000	0.064	0.086	0.241	0.232	0.565	0.053	0.058	0.561	0.564	0.564	1.992	1.158	1.008	0.996	1.008	1.319	1.209	1.313	1.190	1.182
LEIAMA	1.078	1.024	1.546	0.550	0.525	0.563	0.410	1.015	0.087	0.000	0.086	0.275	0.088	0.247	0.086	0.087	0.094	0.013	0.248	0.239	0.570	0.085	0.088	0.565	0.568	0.568	2.023	1.164	1.028	1.006	1.018	1.321	1.216	1.319	1.193	1.202
LEIEAE	1.093	1.042	1.567	0.570	0.532	0.578	0.417	1.044	0.055	0.086	0.000	0.277	0.040	0.243	0.054	0.054	0.047	0.085	0.243	0.237	0.585	0.022	0.040	0.583	0.581	0.580	2.036	1.195	1.034	1.020	1.031	1.353	1.245	1.338	1.229	1.213
LEIENR	1.095	1.047	1.549	0.587	0.557	0.592	0.451	1.040	0.271	0.276	0.278	0.000	0.278	0.298	0.267	0.269	0.276	0.276	0.298	0.289	0.600	0.274	0.277	0.601	0.596	0.594	2.021	1.189	1.039	1.021	1.038	1.345	1.229	1.338	1.215	1.199
LEIGER	1.090	1.037	1.554	0.563	0.529	0.571	0.416	1.033	0.058	0.089	0.040	0.277	0.000	0.243	0.058	0.057	0.032	0.087	0.244	0.237	0.577	0.039	0.017	0.575	0.575	0.573	2.041	1.188	1.032	1.016	1.031	1.349	1.234	1.338	1.219	1.206

Abbrev	ANGDEA	ANGDES	BODSAL	CRIACA	CRIBOM	CRIFAS	ENDMOT	HERMUS	HU-UFS14	LEIAMA	LEIEAE	LEIENR	LEIGER	LEIBRA	LEIDON	LEIINF	LEIMAJ	LEIMEX	LEIPAN	LEIPER	LEPPYR	LEITRO	LEITUR	LOTPAS	LVH60	LVH60a	PERSPE	PHYSPE	STRGAL	STRCUL	STRONC	TRYBRU	TRYCRU	TRYEQU	TRYGRA	TRYRAN
LEIBRA	1.083	1.027	1.539	0.571	0.539	0.580	0.427	1.033	0.241	0.246	0.242	0.296	0.243	0.000	0.240	0.240	0.248	0.247	0.014	0.003	0.586	0.240	0.242	0.587	0.587	0.586	1.997	1.174	1.020	1.010	1.021	1.333	1.225	1.329	1.210	1.190
LEIDON	1.064	1.016	1.531	0.545	0.514	0.560	0.402	1.011	0.004	0.087	0.054	0.267	0.058	0.241	0.000	0.004	0.064	0.086	0.241	0.231	0.564	0.053	0.058	0.564	0.563	0.563	2.008	1.161	1.009	0.997	1.010	1.322	1.209	1.313	1.195	1.192
LEIINF	1.060	1.007	1.523	0.543	0.511	0.556	0.403	1.003	0.000	0.087	0.054	0.267	0.057	0.240	0.004	0.000	0.064	0.085	0.240	0.231	0.562	0.053	0.057	0.559	0.560	0.559	1.974	1.156	1.003	0.993	1.004	1.311	1.203	1.308	1.188	1.178
LEIMAJ	1.068	1.004	1.533	0.546	0.516	0.560	0.404	1.004	0.064	0.094	0.047	0.273	0.032	0.246	0.064	0.064	0.000	0.093	0.247	0.237	0.563	0.046	0.032	0.559	0.563	0.562	1.972	1.159	1.009	0.995	1.008	1.313	1.210	1.309	1.188	1.177
LEIMEX	1.067	1.012	1.531	0.548	0.518	0.558	0.406	1.011	0.086	0.013	0.085	0.274	0.087	0.247	0.085	0.085	0.093	0.000	0.247	0.239	0.567	0.083	0.087	0.561	0.564	0.563	1.979	1.162	1.001	0.996	1.001	1.318	1.209	1.315	1.189	1.182
LEIPAN	1.091	1.032	1.544	0.574	0.540	0.584	0.430	1.038	0.242	0.248	0.243	0.297	0.243	0.014	0.241	0.241	0.249	0.248	0.000	0.014	0.591	0.241	0.242	0.589	0.588	0.586	2.011	1.175	1.029	1.014	1.026	1.333	1.229	1.332	1.210	1.198
LEIPER	1.026	0.994	1.449	0.519	0.504	0.523	0.393	0.962	0.225	0.231	0.227	0.277	0.227	0.003	0.223	0.224	0.229	0.231	0.013	0.000	0.528	0.226	0.227	0.527	0.527	0.526	1.998	1.091	0.976	0.972	0.981	1.231	1.127	1.220	1.122	1.124
LEPPYR	1.080	1.026	1.562	0.330	0.309	0.381	0.658	1.023	0.569	0.572	0.584	0.598	0.577	0.591	0.564	0.567	0.572	0.572	0.593	0.561	0.000	0.580	0.576	0.343	0.365	0.365	2.045	1.166	1.010	1.007	1.014	1.347	1.245	1.338	1.225	1.202
LEITRO	1.085	1.037	1.562	0.564	0.529	0.573	0.411	1.033	0.054	0.085	0.022	0.273	0.039	0.241	0.053	0.053	0.046	0.084	0.242	0.235	0.580	0.000	0.039	0.576	0.575	0.573	2.042	1.191	1.030	1.012	1.027	1.352	1.241	1.329	1.220	1.205
LEITUR	1.085	1.036	1.559	0.562	0.529	0.573	0.415	1.037	0.058	0.088	0.040	0.277	0.017	0.242	0.058	0.057	0.032	0.087	0.243	0.237	0.576	0.039	0.000	0.576	0.574	0.573	2.036	1.187	1.033	1.020	1.027	1.348	1.231	1.338	1.220	1.207
LOTPAS	1.071	1.015	1.561	0.318	0.304	0.373	0.659	1.023	0.567	0.568	0.583	0.601	0.576	0.592	0.564	0.566	0.565	0.568	0.594	0.560	0.343	0.577	0.576	0.000	0.364	0.364	2.032	1.178	1.013	1.002	1.016	1.337	1.234	1.324	1.220	1.198
LVH60	1.042	0.989	1.526	0.327	0.324	0.012	0.645	0.990	0.560	0.562	0.572	0.583	0.566	0.583	0.555	0.557	0.562	0.563	0.582	0.552	0.360	0.566	0.565	0.357	0.000	0.000	1.968	1.155	0.982	0.980	0.989	1.318	1.205	1.318	1.182	1.167
LVH60a	1.043	0.992	1.532	0.326	0.324	0.012	0.645	0.993	0.563	0.563	0.572	0.584	0.567	0.582	0.557	0.559	0.562	0.563	0.582	0.551	0.361	0.567	0.565	0.359	0.000	0.000	1.973	1.157	0.983	0.981	0.992	1.324	1.208	1.324	1.185	1.168
PERSPE	2.048	1.989	2.017	2.052	2.026	2.005	2.040	2.029	2.037	2.036	2.033	2.021	2.040	2.030	2.024	2.016	2.022	2.024	2.032	1.995	2.049	2.042	2.036	2.032	2.012	2.016	0.000	2.057	2.051	2.028	2.060	2.034	1.979	2.042	1.972	2.015
PHYSPE	1.156	1.127	1.583	1.167	1.128	1.169	1.192	1.048	1.172	1.169	1.195	1.187	1.188	1.183	1.165	1.168	1.176	1.172	1.180	1.137	1.167	1.190	1.185	1.177	1.169	1.172	2.051	0.000	1.110	1.106	1.118	1.351	1.258	1.344	1.256	1.249
STRGAL	0.837	0.799	1.481	1.002	0.994	0.996	1.065	0.999	1.021	1.032	1.036	1.038	1.033	1.028	1.010	1.016	1.023	1.022	1.035	1.003	1.011	1.031	1.033	1.013	0.996	0.996	2.050	1.110	0.000	0.265	0.190	1.263	1.167	1.257	1.148	1.148
STRCUL	0.860	0.814	1.469	0.990	0.986	0.985	1.050	0.986	1.003	1.009	1.019	1.021	1.015	1.017	0.997	1.001	1.008	1.007	1.020	0.992	1.007	1.011	1.016	1.000	0.992	0.991	2.025	1.106	0.265	0.000	0.280	1.251	1.146	1.241	1.132	1.144
STRONC	0.846	0.807	1.467	1.002	1.006	0.992	1.068	0.996	1.019	1.023	1.031	1.037	1.031	1.031	1.011	1.016	1.020	1.014	1.034	1.003	1.016	1.026	1.027	1.016	0.999	1.000	2.060	1.118	0.190	0.281	0.000	1.259	1.160	1.251	1.147	1.151
TRYBRU	1.309	1.253	1.548	1.327	1.286	1.323	1.365	1.250	1.333	1.324	1.349	1.339	1.348	1.340	1.321	1.323	1.330	1.330	1.338	1.282	1.345	1.345	1.345	1.333	1.331	1.331	2.026	1.349	1.257	1.249	1.258	0.000	0.810	0.006	0.781	0.806
TRYCRU	1.197	1.154	1.492	1.222	1.163	1.220	1.244	1.141	1.213	1.214	1.236	1.223	1.228	1.226	1.204	1.208	1.217	1.212	1.227	1.167	1.236	1.232	1.226	1.228	1.213	1.212	1.958	1.248	1.161	1.143	1.155	0.807	0.000	0.802	0.542	0.339
TRYEQU	1.300	1.254	1.549	1.326	1.277	1.322	1.361	1.248	1.327	1.324	1.339	1.337	1.338	1.336	1.315	1.319	1.324	1.327	1.337	1.281	1.338	1.329	1.339	1.322	1.330	1.330	2.041	1.346	1.255	1.242	1.251	0.006	0.805	0.000	0.778	0.805
TRYGRA	1.194	1.146	1.494	1.211	1.160	1.207	1.238	1.125	1.202	1.195	1.229	1.213	1.219	1.220	1.196	1.202	1.208	1.205	1.216	1.162	1.226	1.219	1.220	1.220	1.194	1.195	1.972	1.258	1.148	1.133	1.147	0.784	0.549	0.778	0.000	0.536
TRYRAN	1.203	1.161	1.475	1.197	1.157	1.179	1.226	1.120	1.197	1.205	1.213	1.196	1.205	1.198	1.192	1.191	1.195	1.194	1.204	1.159	1.203	1.202	1.204	1.196	1.176	1.176	2.006	1.248	1.146	1.143	1.150	0.808	0.341	0.804	0.535	0.000

Appendix Table 7. GenBank accession numbers of whole-genome sequence data disclosed in this work

Sample name	BioProject	Biosample	Organism	Tax ID	Genome submission	Genome assembly*	SRA study	SRA accession
HU-UFS14	PRJNA398352	SAMN07508840	*Leishmania infantum*	5671	SUB2981723	NSCO00000000	SRP116138	SRR5973233
LVH60	PRJNA398352	SAMN07508845	Trypanosomatidae sp.	1737640	SUB2982025	NSCT00000000	SRP116138	SRR5973230
LVH60a	PRJNA398352	SAMN07508846	Trypanosomatidae sp.	1737640	SUB2982052	NSCU00000000	SRP116138	SRR5973228

*These Whole Genome Shotgun project has been deposited at DDBJ/ENA/GenBank under the listed accessions. The versions described in this work are version XXXX01000000, where X indicates the 4-letter code from each deposited sample.

Appendix Table 8. Available and deduced coding sequences (CDS) for clinical isolates and kinetoplastid species used in this work. All processed sequence data of clinical isolates compared to trypanosomatids reference genomes were organized in a hyperlinked Excel spreadsheet.

Abbreviation	Species name	No. CDS	Median protein size, aa	Average size, aa	Smallest Protein, aa	Largest Protein, aa	Local genome assembly?	Local CDS extraction?
HU-UFS14 (VL)	*Leishmania infantum*	8,234	836	623	50	7,009	Yes	Yes
LVH60 (VL-like)	*Crithidia*-like	10,090	808	627	50	6,937	Yes	Yes
LVH60a (DPSL VL-like)	*Crithidia*-like	10,174	815	631	50	6,937	Yes	Yes
ANGDEA (MX)	*Angomonas deanei*	6,255	682	545	68	6,631	No	Yes
ANGDES (MX)	*Angomonas desouzai*	6,282	547	441	68	6,494	No	Yes
BODSAL (FL)	*Bodo saltans*	17,840	753	548	67	10,369	No	No
CRIACA (MX)	*Crithidia acanthocephali*	11,800	734	483	67	7,926	No	Yes
CRIMEL (MX)	*Crithidia mellificae*	7,660	703	551	78	6,688	No	Yes
CRIFAS (MX)	*Crithidia fasciculata*	13,212	728	509	60	7,313	No	Yes
CRIBOM (MX)	*Crithidia bombi*	7,675	743	574	84	6,938	No	Yes
ENDMOT (DX)	*Endotrypanum monterogeii*	8,291	813	603	69	6,937	No	Yes
HERMUS (MX)	*Herpetomonas muscarum*	10,297	665	447	67	5,610	No	Yes
LEIAET (DX)	*Leishmania aethiopica*	8,485	834	604	67	6,794	No	Yes
LEIAMA (DX)	*Leishmania amazonensis*	7,316	812	620	57	7,572	No	No
LEIBRA (DX)	*Leishmania braziliensis*	8,151	839	624	51	6,733	No	No
LEIDON (DX)	*Leishmania donovani*	7,960	840	618	50	7,009	No	No
LEIENR (DX)	*Leishmania enrietti*	8,092	812	607	69	7,009	No	Yes
LEIGER (DX)	*Leishmania gerbilli*	8,570	833	603	69	7,029	No	Yes
LEIINF (DX)	*Leishmania infantum*	8,141	843	632	50	7,009	No	No
LEIMAJ (DX)	*Leishmania major*	8,306	836	630	51	17,392	No	No
LEIMEX(DX)	*Leishmania mexicana*	8,137	842	629	51	6,738	No	No
LEIPAN (DX)	*Leishmania panamensis*	7,742	835	625	51	6,732	No	No
LEIPER (DX)	*Leishmania peruviana*	7,155	712	537	70	6,902	No	Yes
LEITRO (DX)	*Leishmania tropica*	8,598	841	609	70	7,028	No	Yes
LEITUR (DX)	*Leishmania turanica*	8,465	847	611	67	7,032	No	Yes
LEPPYR (MX)	*Leptomonas pyrrhocoris*	9,284	845	616	50	7,638	No	No
LOTPAS (MX)	*Lotmaria passim*	12,838	760	475	67	7594	No	No
PERSPE (EN)	*Perkinsela sp.*	4,850	475	360	67	4,342	Yes	Yes
PHYSPE (DX)	*Phytomonas sp.*	6,410	626	458	50	6,650	No	No
STRGAL (MX)	*Strigomonas galati*	7,329	645	517	69	7,053	No	Yes
STRCUL (MX)	*Strigomonas culicis*	6,785	645	514	69	6,413	No	Yes
STRONC (MX)	*Strigomonas oncopelti*	6,778	646	513	72	7,053	No	Yes
TRYBRU (DX)	*Trypanosoma brucei*	9,642	648	455	50	6,613	No	No
TRYCON (DX)	*Trypanosoma congolense*	5,872	451	376	99	4,553	No	No
TRYCRU (DX)	*Trypanosoma cruzi*	19,062	661	506	50	4,848	No	No
TRYEQU (DX)	*Trypanosoma equiperdum*	7,659	634	499	67	4,972	No	No
TRYGRA (DX)	*Trypanosoma grayi*	10,576	593	435	66	4,982	No	No

TRYRAN (DX)	*Trypanosoma rangeli*	7,331	588	464	68	4,449	No	No

CDS, coding sequence; DPSL, disseminated popular skin lesions; DX, dixenous (2-hosts) life cycle infecting both vertebrates and invertebrates; EN, endosymbiont; FL, free-living nonparasitic; MX: monoxenous (1-host) life cycle only in invertebrates.

Appendix Table 9. Accession numbers of small subunit rRNA (SSU rRNA) and glyceraldehyde 3-phosphate dehydrogenase gene (GAPDH) sequences used in phylogenetic analysis

Appendix Figure 1 (SSU rRNA): sequences retrieved from NCBI	
Crithidia bombi	KM980185.1
Crithidia fasciculata	Y00055.1
Crithidia luciliae thermophila	KY264937.1
Endotrypanum colombiensis	KX790768.1
Endotrypanum monterogeii	X53911.1
Leishmania amazonensis	JX030083.1
Leishmania aethiopica	M81428.1
Leishmania braziliensis	JX030135.1
Leishmania chagasi	M81430.1
Leishmania donovani	XR002966730.1
Leishmania enriettii	KX790777.1
Leishmania infantum	XR001203206.1
Leishmania major	XR002460813.1
Leishmania mexicana	GQ332360.1
Leishmania panamensis	JN003595.1
Leishmania tropica	GQ332363.1
Leptomonas pyrrhocoris	XR001548753.1
Leptomonas seymouri	AF153040.2
Lotmaria passim	KM980188.1
Trypanosoma brucei	XR002989632.1
Trypanosoma cruzi	AY785570.1
Trypanosoma equiperdum	AJ223564.1
Trypanosoma grayi	AJ005278.1
Trypanosoma rangeli	AY491753.1
Appendix Figure 2 (SSU rRNA): sequences generated in this work	
LVH60	LT990192*
LVH60a	LT990193*
HU-UFS14	LT990187*
LVH60 clo.1	PRJEB33751*
LVH60 clo.2	PRJEB33751*
LVH60 clo.3	PRJEB33751*
LVH60 clo.4	PRJEB33751*
LVH60 clo.5	PRJEB33751*
LVH60a clo.1	PRJEB33751*
LVH60a clo.2	PRJEB33751*
LVH60a clo.3	PRJEB33751*
Appendix Figure 3 (GAPDH): sequences retrieved from NCBI	
Crithidia bombi	ADI58767.1
Crithidia fasciculata	AAD02465.1
Crithidia thermophila	ARH02611.1
Endotrypanum colombiensis	KX790711.1
Endotrypanum monterogeii	APQ47645.1
Leishmania braziliensis	XP_001566920.1
Leishmania chagasi	AHG99347.1
Leishmania donovani	XP_003863010.1
Leishmania enriettii	APQ47654.1
Endotrypanum equatorensis	KX790718.1
Leishmania infantum	XP_001467145.1
Leishmania major	XP_001684904.1
Leishmania mexicana	XP_003877441.1
Leishmania panamensis	XP_010701314.1
Leptomonas pyrrhocoris	AEX31182.1
Leptomonas seymouri	AF047495.1
Lotmaria passim	AIF30826.1
Trypanosoma brucei	CAF04248.1
Trypanosoma cruzi	RNC47212.1
Trypanosoma grayi	CAF04222.1
Trypanosoma rangeli	ADD83084.1
Appendix Figure 3 (GAPDH): sequences generated in this work	

LVH60	LVH60–129333 851†
LVH60a	LVH60a-124042 1044†
HU-UFS14	HU-66665 3†
LVH60 clo.2	PRJEB33769
LVH60 clo.5	PRJEB33769
LVH60a clo.2	PRJEB33769
HUUFS14 clo.2	PRJEB33769
HUUFS14 clo.3	PRJEB33769

*Sequences were deposited in European Nucleotide Archive (ENA) under these accession numbers and are available at http://www.ebi.ac.uk/ena/data/view/.
†Translated in-house from genomic data. Protein sequences from WGS data were compiled and provided as .zip file available at http://exon.niaid.nih.gov/transcriptome/Cridia/fasta.zip by request.

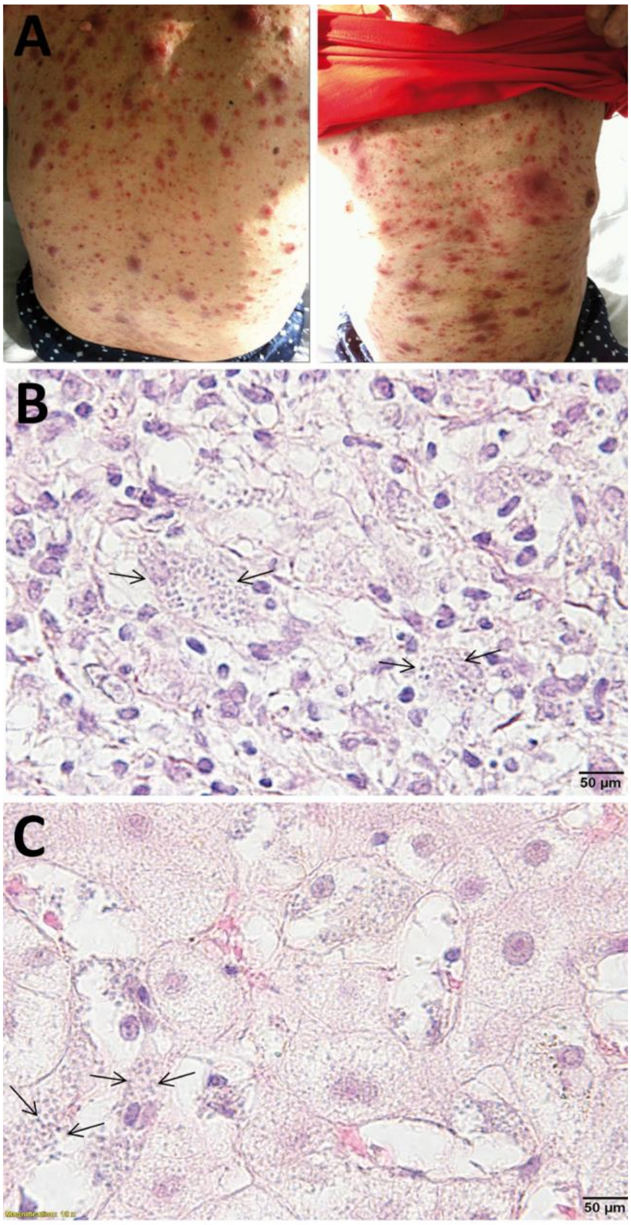

Appendix Figure 1. Fatal case of visceral leishmaniasis-like illness. Disseminated papular and nodular skin lesions (DPSL) after 8 months of treatment with liposomal amphotericin B (A).

Representative photomicrographs of amastigotes in sections of skin (B) and liver (C) from the infected patient. H&E staining is shown at a magnification of 1,000, and the scale bar represents 50 µm. Arrows indicate multiple amastigotes within the macrophages.

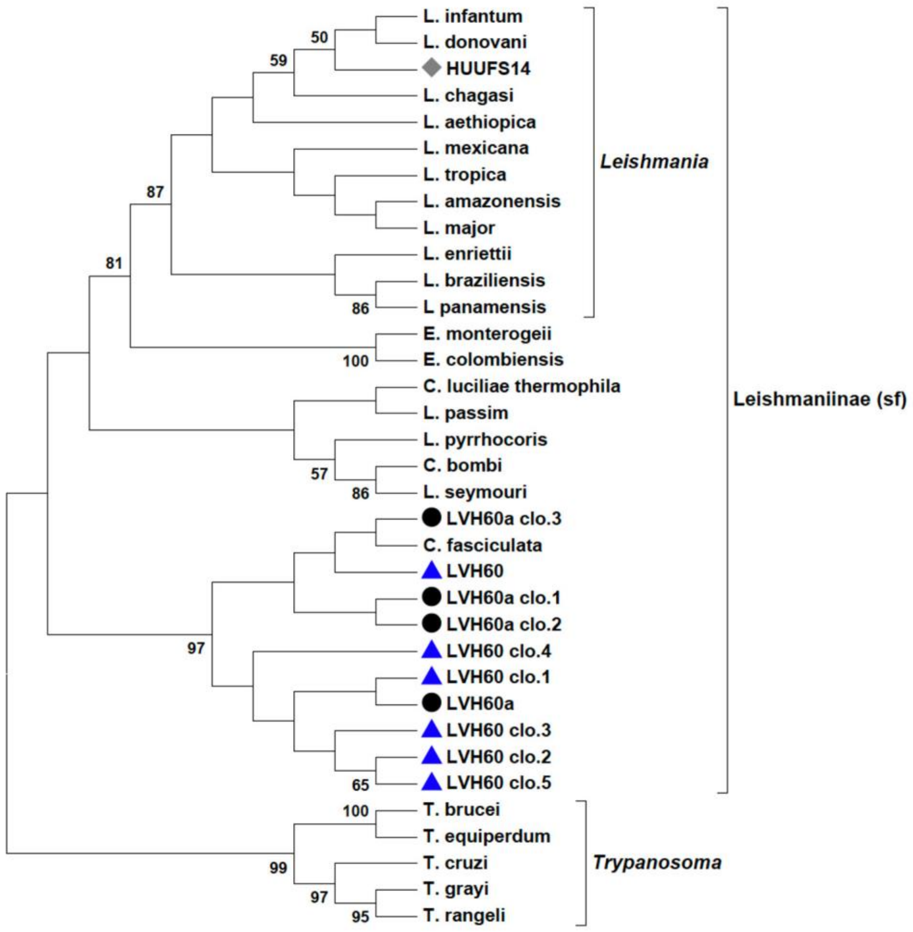

Appendix Figure 2. Phylogenetic analysis of small subunit ribosomal (SSU rRNA) sequences of clinical isolates LVH60, LVH60a and HUUFS14. Phylogenetic tree was constructed using the maximum-likelihood (ML) method based on the Tamura-Nei model, using nucleotide sequences obtained from NBCI (access numbers available in Appendix Table 9). The numbers next to the branches represent the percentage of replicate trees in which the associated taxa group in the bootstrap test (1,000 replicates). All positions with <50% site coverage, containing missing gaps and data, have been deleted. The initial tree for the heuristic search was obtained by applying the neighbor-joining (NJ) method to an array of estimated peer distances using the maximum composite likelihood (MCL) approach. The analysis involved 35 aa sequences and a total of 674 positions in the final dataset. The gray diamond indicates a reference laboratory *L. infantum* strain (control). Black circles indicate parasite strains isolated from the skin lesion, and the blue triangles indicate parasite strains isolated from the bone marrow, both clinical isolates come from an atypical fatal case of LV of a patient called LVH60. Clo.#: clonal colonies for respective parasite strains; sf: subfamily.

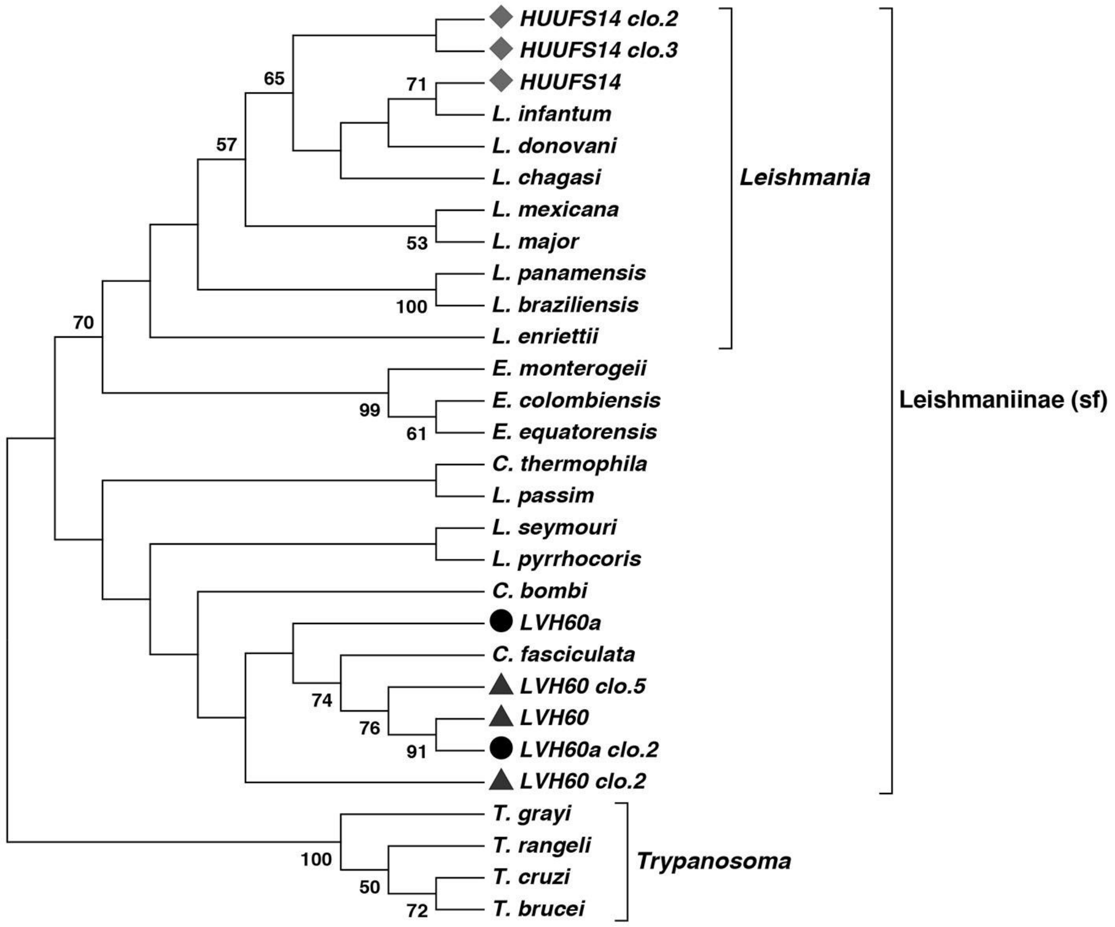

Appendix Figure 3. Phylogenetic analysis of gGAPDH (glycosomal glyceraldehyde 3-phosphate dehydrogenase) protein sequences from LVH60, LVH60a and HU-UFS14 clinical: Phylogenetic tree was constructed using the maximum-likelihood (ML) method based on the JTT matrix replacement model with sequences translated using the ORF finder (NCBI) tools and the analysis involved 21 aa sequences obtained through the NCBI (accession numbers in Appendix Table 9) and 8 sequences from clinical isolates. A total of 294 positions in the final dataset. Numbers next to the branches represent the percentage of replicate trees in which the associated taxa group in the bootstrap test (1,000 replicates). The initial tree for the heuristic search was obtained by applying the neighbor-joining method to an array of estimated peer distances using a JTT model. The gray diamond indicates a reference laboratory of *L. infantum* strain (control). Black circles indicate strains of parasites isolated from the skin lesion, and blue triangles indicate strains of parasites isolated from bone marrow, both clinical isolates come from an atypical LV fatal case of a patient named LVH60. Clo #: clonal colonies for their parasitic strains; sf: subfamily.

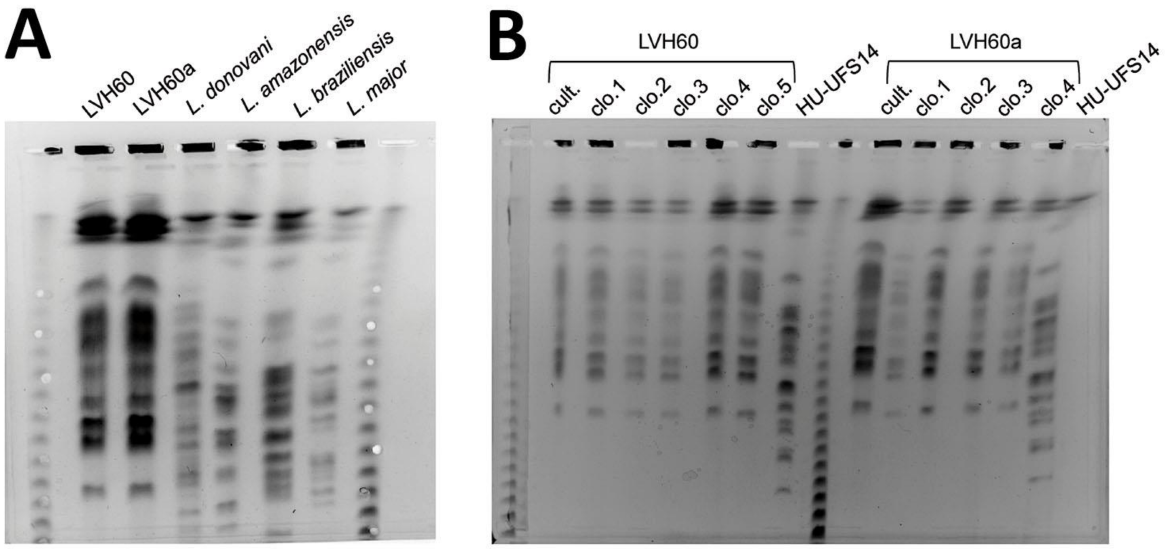

Appendix Figure 4. Molecular karyotyping of LVH60 and LVH60a clinical isolates by pulsed-field gel electrophoresis (PFGE). Ethidium bromide-stained pattern of chromosomal DNA bands from promastigote cultures of LVH60, LVH60a, *L. donovani* Bob strain (MHOM/SD/62/1S-CL2D), *L. amazonensis* (MHOM/BR/73/2269), *L. braziliensis* (MHOM/BR/1975/M2903), *L. major* (MRHO/SU/59/P/LV39) after separation by PFGE (A) and from PFGE of LVH60 and LVH60a primary cultures (cult.) and their respective clones (clo.#) (B). HU-UFS14 (*L. infantum*) was used as a reference control strain.

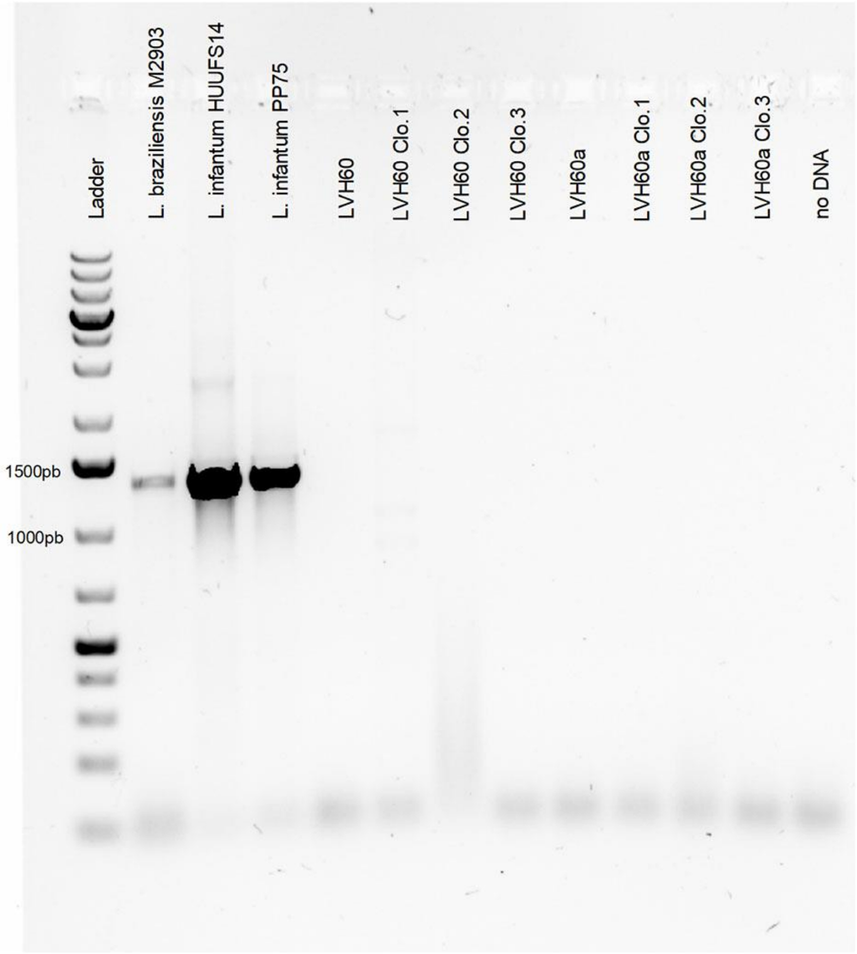

Appendix Figure 5. DNA electrophoresis in 1% agarosel gel of PCR amplicons from HSP70 gene of *Leishmania*. Genomic DNA from *L. braziliensis* (MHOM/BR/1975/M2903), *L. infantum* (HUUFS14 laboratory strain) and *L. infantum* (MHOM/BR/74/PP75) strains were used as positive control. Negative control for PCR reactions was performed without DNA template (no DNA). LVH60 is the parasite strain isolated from bone marrow, and LVH60a is the parasite strain isolated from skin lesion. Clo.#: clonal colonies for respective parasite strains. Expected amplicon size using primers described elsewhere (*16*) is 1,286 pb. Molecular size marker: GeneRuler 1 kb Plus DNA Ladder (Thermo Scientific).

References

1. Nascimento MSL, Carregaro V, Lima-Júnior DS, Costa DL, Ryffel B, Duthie MS, et al. Interleukin 17A acts synergistically with interferon γ to promote protection against *Leishmania infantum* infection. J Infect Dis. 2014;jiu531. PubMed

2. Nascimento MSL, Ferreira MD, Quirino GFS, Maruyama SR, Krishnaswamy JK, Liu D, et al. NOD2-RIP2–mediated signaling helps shape adaptive immunity in visceral leishmaniasis. J Infect Dis. 2016;214:1647–57. PubMed https://doi.org/10.1093/infdis/jiw446

3. Quirino GFS, Nascimento MSL, Davoli-Ferreira M, Sacramento LA, Lima MHF, Almeida RP, et al. Interleukin-27 (IL-27) mediates susceptibility to visceral leishmaniasis by suppressing the IL-17-neutrophil response. Infect Immun. 2016;84:2289–98. PubMed https://doi.org/10.1128/IAI.00283-16

4. Lima MHF, Sacramento LA, Quirino GFS, Ferreira MD, Benevides L, Santana AKM, et al. *Leishmania infantum* parasites subvert the host inflammatory response through the adenosine $A2_A$ receptor to promote the establishment of infection. Front Immunol. 2017;8:815. PubMed https://doi.org/10.3389/fimmu.2017.00815

5. Sacramento LA, da Costa JL, de Lima MH, Sampaio PA, Almeida RP, Cunha FQ, et al. Toll-like receptor 2 is required for inflammatory process development during *Leishmania infantum* infection. Front Microbiol. 2017;8:262. PubMed https://doi.org/10.3389/fmicb.2017.00262

6. Cupolillo E, Grimaldi G Jr, Momen H. A general classification of New World *Leishmania* using numerical zymotaxonomy. Am J Trop Med Hyg. 1994;50:296–311. PubMed https://doi.org/10.4269/ajtmh.1994.50.296

7. Noyes HA, Stevens JR, Teixeira M, Phelan J, Holz P. A nested PCR for the ssrRNA gene detects *Trypanosoma binneyi* in the platypus and *Trypanosoma* sp. in wombats and kangaroos in Australia. Int J Parasitol. 1999;29:331–9. PubMed https://doi.org/10.1016/S0020-7519(98)00167-2

8. Schönian G, Nasereddin A, Dinse N, Schweynoch C, Schallig HDFH, Presber W, et al. PCR diagnosis and characterization of *Leishmania* in local and imported clinical samples. Diagn Microbiol Infect Dis. 2003;47:349–58. PubMed https://doi.org/10.1016/S0732-8893(03)00093-2

9. Borghesan TC, Ferreira RC, Takata CSA, Campaner M, Borda CC, Paiva F, et al. Molecular phylogenetic redefinition of *Herpetomonas* (Kinetoplastea, Trypanosomatidae), a genus of insect parasites associated with flies. Protist. 2013;164:129–52. PubMed https://doi.org/10.1016/j.protis.2012.06.001

10. Da Silva FM, Noyes H, Campaner M, Junqueira ACV, Coura JR, Añez N, et al. Phylogeny, taxonomy and grouping of *Trypanosoma rangeli* isolates from man, triatomines and sylvatic mammals from widespread geographical origin based on SSU and ITS ribosomal sequences. Parasitology. 2004;129:549–61. PubMed https://doi.org/10.1017/S0031182004005931

11. Teixeira MMG, Borghesan TC, Ferreira RC, Santos MA, Takata CSA, Campaner M, et al. Phylogenetic validation of the genera *Angomonas* and *Strigomonas* of trypanosomatids harboring bacterial endosymbionts with the description of new species of trypanosomatids and of proteobacterial symbionts. Protist. 2011;162:503–24. PubMed https://doi.org/10.1016/j.protis.2011.01.001

12. Fraga J, Montalvo AM, De Doncker S, Dujardin J-C, Van der Auwera G. Phylogeny of *Leishmania* species based on the heat-shock protein 70 gene. Infect Genet Evol. 2010;10:238–45. PubMed https://doi.org/10.1016/j.meegid.2009.11.007

13. Schönian G, Mauricio I, Cupolillo E. Is it time to revise the nomenclature of *Leishmania*? Trends Parasitol. 2010;26:466–9. PubMed https://doi.org/10.1016/j.pt.2010.06.013

14. Espinosa OA, Serrano MG, Camargo EP, Teixeira MMG, Shaw JJ. An appraisal of the taxonomy and nomenclature of trypanosomatids presently classified as *Leishmania* and *Endotrypanum*. Parasitology. 2018;145:430–42. PubMed https://doi.org/10.1017/S0031182016002092

15. Kostygov AY, Yurchenko V. Revised classification of the subfamily Leishmaniinae (Trypanosomatidae). Folia Parasitol (Praha). 2017 Jul 10;64.

16. Espada CR, Ortiz PA, Shaw JJ, Barral AMP, Costa JML, Uliana SRB, et al. Identification of *Leishmania* (*Viannia*) species and clinical isolates of *Leishmania* (*Leishmania*) *amazonensis* from Brazil using PCR-RFLP of the heat-shock protein 70 gene reveals some unexpected observations. Diagn Microbiol Infect Dis. 2018;91:312–8. PubMed https://doi.org/10.1016/j.diagmicrobio.2018.03.004

17. Cruz A, Beverley SM. Gene replacement in parasitic protozoa. Nature. 1990;348:171–3. PubMed https://doi.org/10.1038/348171a0

18. de Oliveira JPC, Fernandes F, Cruz AK, Trombela V, Monteiro E, Camargo AA, et al. Genetic diversity of *Leishmania amazonensis* strains isolated in northeastern Brazil as revealed by DNA sequencing, PCR-based analyses and molecular karyotyping. Kinetoplastid Biol Dis. 2007;6:5. PubMed https://doi.org/10.1186/1475-9292-6-5

19. Titus RG, Marchand M, Boon T, Louis JA. A limiting dilution assay for quantifying *Leishmania major* in tissues of infected mice. Parasite Immunol. 1985;7:545–55. PubMed https://doi.org/10.1111/j.1365-3024.1985.tb00098.x

20. Buffet PA, Sulahian A, Garin YJ, Nassar N, Derouin F. Culture microtitration: a sensitive method for quantifying *Leishmania infantum* in tissues of infected mice. Antimicrob Agents Chemother. 1995;39:2167–8. PubMed https://doi.org/10.1128/AAC.39.9.2167

21. Simpson JT, Wong K, Jackman SD, Schein JE, Jones SJ, Birol I. ABySS: a parallel assembler for short read sequence data. Genome Res. 2009;19:1117–23. PubMed https://doi.org/10.1101/gr.089532.108

22. Rice P, Longden I, Bleasby A. EMBOSS: the European Molecular Biology Open Software Suite. Trends Genet. 2000;16:276–7. PubMed https://doi.org/10.1016/S0168-9525(00)02024-2

23. Altschul SF, Madden TL, Schäffer AA, Zhang J, Zhang Z, Miller W, et al. Gapped BLAST and PSI-BLAST: a new generation of protein database search programs. Nucleic Acids Res. 1997;25:3389–402. PubMed https://doi.org/10.1093/nar/25.17.3389

24. O'Leary NA, Wright MW, Brister JR, Ciufo S, Haddad D, McVeigh R, et al. Reference sequence (RefSeq) database at NCBI: current status, taxonomic expansion, and functional annotation. Nucleic Acids Res. 2016;44(D1):D733–45. PubMed https://doi.org/10.1093/nar/gkv1189

25. Bateman A, Martin MJ, O'Donovan C, Magrane M, Alpi E, Antunes R, et al.; The UniProt Consortium. UniProt: the universal protein knowledgebase. Nucleic Acids Res. 2017;45(D1):D158–69. PubMed https://doi.org/10.1093/nar/gkw1099

26. Wall DP, Deluca T. Ortholog detection using the reciprocal smallest distance algorithm. Methods Mol Biol. 2007;396:95–110. PubMed https://doi.org/10.1007/978-1-59745-515-2_7

27. Suzuki R, Shimodaira H. Pvclust: an R package for assessing the uncertainty in hierarchical clustering. Bioinformatics. 2006;22:1540–2. PubMed https://doi.org/10.1093/bioinformatics/btl117

28. Jones DT, Taylor WR, Thornton JM. The rapid generation of mutation data matrices from protein sequences. Comput Appl Biosci. 1992;8:275–82. PubMed https://doi.org/10.1093/bioinformatics/8.3.275

29. Tamura K, Stecher G, Peterson D, Filipski A, Kumar S. MEGA6: Molecular Evolutionary Genetics Analysis version 6.0. Mol Biol Evol. 2013;30:2725–9. PubMed https://doi.org/10.1093/molbev/mst197
